# Canvassing Prospects of Glyco-Nanovaccines for Developing Cross-Presentation Mediated Anti-Tumor Immunotherapy

**DOI:** 10.3390/vaccines10122049

**Published:** 2022-11-30

**Authors:** Amina I. Makandar, Mannat Jain, Eiji Yuba, Gautam Sethi, Rajesh Kumar Gupta

**Affiliations:** 1 Protein Biochemistry Research Centre, Dr. D. Y. Patil Biotechnology & Bioinformatics Institute, Dr. D. Y. Patil Vidyapeeth, Tathawade, Pune 411033, Maharashtra, India; 2 Department of Applied Chemistry, Graduate School of Engineering, Osaka Metropolitan University, 1-1 Gakuen-cho, Naka-ku, Sakai 599-8531, Osaka, Japan; 3 Department of Pharmacology, Yong Loo Lin School of Medicine, National University of Singapore, Singapore 117600, Singapore

**Keywords:** lectin, galectin, vaccine, antigen cross-presentation, dendritic cells, C-type lectin receptors, glycan, CD8^+^ T lymphocytes, cancer immunotherapy

## Abstract

In view of the severe downsides of conventional cancer therapies, the quest of developing alternative strategies still remains of critical importance. In this regard, antigen cross-presentation, usually employed by dendritic cells (DCs), has been recognized as a potential solution to overcome the present impasse in anti-cancer therapeutic strategies. It has been established that an elevated cytotoxic T lymphocyte (CTL) response against cancer cells can be achieved by targeting receptors expressed on DCs with specific ligands. Glycans are known to serve as ligands for C-type lectin receptors (CLRs) expressed on DCs, and are also known to act as a tumor-associated antigen (TAA), and, thus, can be harnessed as a potential immunotherapeutic target. In this scenario, integrating the knowledge of cross-presentation and glycan-conjugated nanovaccines can help us to develop so called ‘glyco-nanovaccines’ (GNVs) for targeting DCs. Here, we briefly review and analyze the potential of GNVs as the next-generation anti-tumor immunotherapy. We have compared different antigen-presenting cells (APCs) for their ability to cross-present antigens and described the potential nanocarriers for tumor antigen cross-presentation. Further, we discuss the role of glycans in targeting of DCs, the immune response due to pathogens, and imitative approaches, along with parameters, strategies, and challenges involved in cross-presentation-based GNVs for cancer immunotherapy. It is known that the effectiveness of GNVs in eradicating tumors by inducing strong CTL response in the tumor microenvironment (TME) has been largely hindered by tumor glycosylation and the expression of different lectin receptors (such as galectins) by cancer cells. Tumor glycan signatures can be sensed by a variety of lectins expressed on immune cells and mediate the immune suppression which, in turn, facilitates immune evasion. Therefore, a sound understanding of the glycan language of cancer cells, and glycan–lectin interaction between the cancer cells and immune cells, would help in strategically designing the next-generation GNVs for anti-tumor immunotherapy.

## 1. Getting Familiar with the Concept of ‘Glyconanovaccine’

### How Did We Get Here?

Cancer cells are the body’s own cells that have altered and deviated from the normal cellular homeostasis and are detrimental to the body, which arise due to the breakdown of the normal cell cycle progression leading to abnormal cell proliferation. Epigenetic changes, disruption in regulatory factors, and the tumor microenvironment (TME) play a significant role in tumor progression and metastasis and, therefore, can be targeted to cure cancer [1,2]. The pursuit to find an effective remedy for cancer has become one of the greatest challenges of medical research. The choice of cancer therapy to opt for depends on the type and development stage of cancer. Treatment of cancer at the primary site in a constrained environment is usually operative; however, cancer in the metastatic stage is difficult to combat and could be terminal [3,4]. The most frequently used conventional cancer strategies to treat metastatic cancer include chemotherapy and radiotherapy. These therapies are used individually or in combination depending on tumor grade and the status of metastasis [5,6]. However, these therapeutic strategies show a lack of specificity and may exhibit adverse effects on patients, causing substantial harm rather than good [7,8].

Conventional chemotherapy generally involves the use of cytotoxic and poorly soluble drugs that target the rapidly dividing cancer cells, conversely affecting healthy rapidly dividing cells, such as hair follicles and mucosa [9,10,11]. Furthermore, chemotherapeutics usually have low circulation half-lives, and a low therapeutic index [12], and may develop systemic toxicity. Furthermore, chemotherapy requires high drug doses to be administered, which may, in turn, cause multidrug resistance (MDR) due to increased efflux pumps (e.g., P-glycoprotein—Pgp—in the cell membrane owing to a decrease in levels of chemotherapeutic accumulation) [13,14,15]. Radiation involves localized treatment wherein ionizing radiation stimulates the death of the tumor cells providing confined control and improved patient survival. Nonetheless, limited efficacy, tolerance of tumors to radiation as compared to the adjoining normal tissue, MDR, obscurity in the target area, and tumor boundaries are the major confronting contraventions [16,17]. In addition, upcoming therapies, such as miRNA therapies are also unreliable as they are prone to degradation by endonucleases [18]. However, using chemotherapy and radiation therapy in combination with immunotherapy utilizing antigen cross-presentation mechanism on dendritic cells (DCs) has been proven to be efficacious in cancer treatment [19]. Another kind of anti-tumor therapy is photothermal therapy, which makes use of photothermal agents to convert the light energy into local hyperthermia for tumor ablation. These therapies include structurally engineered layered metal oxides (PVP-MoO_3−x_ nanobelts) and titanium-based nanoparticles (titanium oxides, titanium carbides, etc.) [20,21]. 

The attempted strategies to at least confine malignancies have been ineffective and are laden with downsides. This is because the process of obtaining the model solution is complex due to the fact that tumors can exploit bodily mechanisms to grow and spread in the body. Moreover, owing to being recognized as ‘self’, the cancer cells sustain insufficient immunogenicity to cast strong cytotoxic immune responses, as the cognate T cells against the tumor-associated antigen (TAA) are eliminated in the central tolerance in the thymus [22]. To provide protection against cancer, the body must activate innate immunity, which produces DCs that can be employed for antigen presentation. It is also essential to activate natural killer (NK) cells and increase the generation of cytotoxic T cells and antibodies. To create significant immunogenicity against cancer cells, it is also necessary to modulate the immunosuppressive TME [8,23,24]. Thus, developing alternative anti-tumor therapeutic approaches is an absolute necessity. Here, so far, overcoming the resistance posed by the immune system of the patient to attack and eliminate tumor is a viable cancer immunotherapy approach. The main benefits of triggering the body’s own defense mechanism against the cancer cells by deploying the knowledge of immune regulators are specificity (distinct Ag elicits a specific immune response), immunologic memory (boosted immune response to recurrent exposures to the same Ag), and adaptability, which thereby help to combat and prevent cancer relapses [25]. The immune responses of cytotoxic T lymphocytes (CTL) mediated through CD8^+^ T cells and NK cells have been reported to recognize and eliminate various solid tumors [26]. 

The DCs are proficient antigen-presenting cells (APCs) that phagocytose the pathogens, and further break them into protein and lipid fragments for the presentation to T lymphocytes; as such, they create a link between innate and adaptive immunity. The pattern recognition receptors (PRRs) present on the DCs are of a particular class of cell surface markers that recognize ligands which are antigenic subunits or molecules present on microbes. Ligands binding to the PRRs stimulate the T cell immunity. Various dendritic cell (DC) PRRs and their respective ligands have been identified. However, only two main PRRs-C-type lectin receptors (CLRs) and Toll-like receptors (TLRs) are known to participate in the antigen recognition process and can be exploited for formulating vaccines aimed at stimulating DC-mediated anti-cancer immunity [27]. Taking these properties of DCs into account, targeting DCs—the most efficient key regulators of the immune system through antigen cross-presentation—via an immune mechanism fabricating cytotoxic immune response may, perhaps, be a potential solution to combat cancer.

Polymeric nanoparticles (NPs) have received a considerable amount of attention and are of great interest in different fields, such as pharmaceuticals and biomedicals, due to their versatile properties and applications [28,29]. One such application is drug delivery [30,31]. These can deliver different drugs and biomolecules, which can be used to specifically target and deliver antigens for immunotherapy against cancer [32]. Many vaccine delivery methods based on polymer particles have been implemented to deliver antigens to DCs; however, because of the absence of pathogen-associated molecular patterns (PAMPs) on these biodegradable polymers, their interaction with DCs is limited [33,34]. Studies on particle-conjugated proteins have been reported that show that they are presented more efficiently than soluble proteins [35]. Carbohydrate-based cell targeting has gained significant attention in the past decade with the foundational knowledge of the ability of the body to recognize the pathogens by identifying exogenous carbohydrates [36]. Combining polymeric particles with glycans can be used to better target the antigens to the DCs without compromising the specificity. 

The DCs display various carbohydrate-recognizing receptors that help uptake antigen (Ag), destining it for immune clearance. Glycans act as ligands for such DC-receptors (DCRs) and, furthermore, glycans are expressed throughout the body; their immunogenicity is below par and, thus, they are ideal for developing DC cross-presentation-mediated immunotherapy [37]. Ushering TAAs directly to such DC-receptors in combination with the glycan-coated NPs may help orchestrate the cytotoxic arm of the immune response against cancer via cross-presentation. This foreseen approach has the potential to effectively eradicate the cancer cells from the body and, hence, it is of profound importance.

## 2. At a Glance 

### 2.1. Antigen Presentation

In humans, T cells can recognize and become activated by antigens only when they are in a bound condition to specialized cell surface proteins referred to as major histocompatibility complexes (MHCs). These MHC molecules exhibit high polymorphism, having multiple allotypes which can be classified on the basis of the difference in the conformation of their peptide-binding grooves. These allotypes are called ‘human lymphocyte antigen (HLA) molecules’, and these can be classified into the HLA-A, HLA-B, and HLA-C. These MHCs are immune surveillance intermediaries that synchronize interactions between lymphocytes and macrophages [38,39]. Cells bearing HLA bound to the antigen are termed as ‘antigen-presenting cells’ (APCs), and the mechanism by which APCs display processed antigen fragments on their surface in the form that is recognized by the lymphocytes is termed as ‘antigen presentation’. The types of antigen presentation are discussed below. 

#### 2.1.1. MHC Class I Antigen Presentation

The MHC class I molecules commonly present peptide antigens derived from endogenously originated proteins (such as viral proteins and cancerous cells). Endogenous antigens are tagged with ubiquitin and, later, various nucleated cells degrade these endogenous antigens, usually with the help of an enzyme complex called proteasome to produce oligopeptides [40,41]. The majority of oligopeptides are subjected to hydrolysis by peptidases to produce amino acid residues. However, a few oligopeptides bypass hydrolysis and are routed to the endoplasmic reticulum (ER) by a specialized transporter, also referred to as transporter associated with antigen processing (TAP). These MHC I-peptide complexes then move through the Golgi complex and are further transported to the surfaces of APCs. These MHC class I-peptide complexes presented to CD8^+^ T cells help the CD8^+^ T cells to identify and clear cancer cells or virus infected cells.

#### 2.1.2. MHC Class II Antigen Presentation

Exogenous antigens are acquired by endocytosis or internalized in APCs to produce peptides. The MHC II-associated proteins are synthesized in the ER. The attachment of the invariant chain (Ii) in the ER lumen stabilizes the MHC class II peptide-binding groove prior to the commencement of the MHC class II pathway of antigen presentation. Further, this invariant chain–MHC class II complex is then directed to the late endosomal compartments collectively known as MHC-II enriched compartments (MIIC) via the trans-Golgi network. These MIICs have characteristics of late endocytic multivesicular bodies (MVBs) or lysosomes. Now, in MIIC, proteolytic degradation of Ii takes place via proteases (especially cathepsin S and L) causing a small fragment called CLIP to remain in the peptide-binding groove. Further, antigens internalized in the APCs are also directed to MIICs for degradation. Antigenic peptides produced in MIICs are directly loaded onto MHC-II, which resides inside the same compartment. Then, HLA-DM, an endosomal chaperon, accelerate CLIP removal, allowing high-affinity antigen-derived peptides to attach to MHC class II molecules [42]. The peptide-bound MHC class II complex can then move to the cell surface of APCs, where antigen presentation takes place. Peptide-bound MHC class II molecules are thereafter displayed to the CD4^+^ T cells. This response is primed by exogenous antigens, which enter the MHC class II pathway via professional APCs [43,44].

## 3. Background to The Subject Matter

### 3.1. Antigen Cross-Presentation

Although MHC class I- and II-associated antigen presentation is typically directed through the two distinct pathways, the routes of these pathways are not entirely parted. In 1976, the pioneering work of Michael John Bevan paved the way to the discovery of cross-presentation [45]. Cross-presentation is an atypical mechanism stirring under some rare circumstances wherein exogenous antigens (e.g., cell debris-associated antigens, tumor cells, and apoptotic cells) are presented in association with a MHC class I molecule, thereby triggering CTL response. Here, the antigens are internalized by DCs as opposed to the expression of antigens observed in conventional MHC class I presentation. The mechanism of uptake and proteolytic degradation of antigens in both cross-presentation and MHC class II antigen presentation is similar. However, during cross-presentation, some of the antigenic fragments escape from the endosomes and move into the cytosol and are further subjected to proteasomal degradation. Cross-presentation is considered not as an outcome of the accidental release of the antigen from the endosomal compartment but rather is a selective intracellular pathway that links the lumen of endocytic compartments to the cytosol [46]. Then, the resultant peptides enter the ER, where they are loaded onto the MHC class I molecules and carried to the cell surface for their presentation to the CD8^+^ T lymphocytes. As a result, it changes the fate of the downstream immune response from classical routing to a cytotoxic immune response.

**Figure 1:**
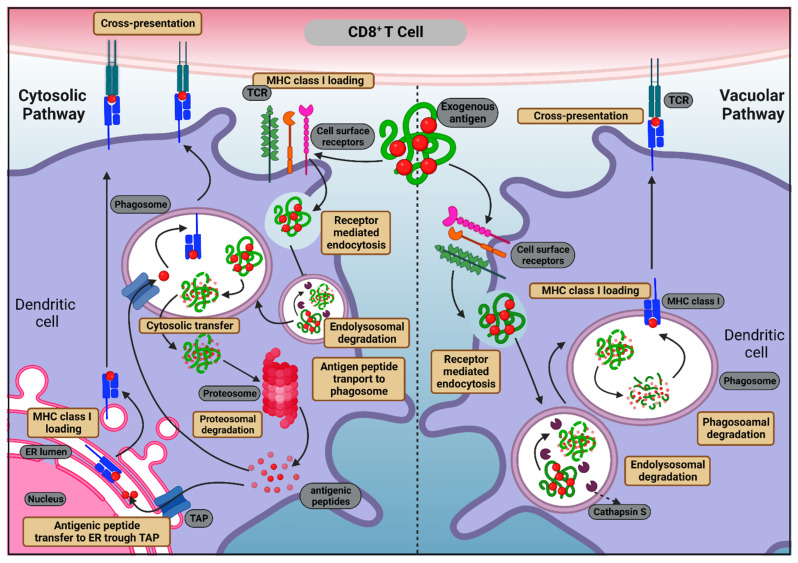
Overview of the cross-presentation pathways in DCs. Cross-presentation of internalized antigens mainly occurs via the following two pathways: (i) the cytosolic pathway and (ii) the vacuolar pathway. Endosomal proteases (cathepsin S) in the vacuolar pathway break-down the internalized antigen into smaller peptides, which are then directly loaded onto MHC class I molecules. The peptide–MHC complex is delivered to the cell surface for CD8^+^ T cell recognition. The exogenous antigen is internalized by endocytosis or phagocytosis in the cytosolic pathway and delivered to the cytosol for proteasomal breakdown to produce shorter antigenic peptides. Furthermore, the antigen-derived peptides are transported to the ER via TAP along with the other ER proteins and then loaded onto the MHC class I molecule in the ER itself. Additionally, TAP is used to deliver the antigenic peptides into the phagosomes, where they are loaded onto the MHC class I molecule and further transferred onto the cell surface for CD8^+^ T cell recognition (Created with BioRender.com accessed on 27 September 2022).

Antigen cross-presentation primarily happens through the following two pathways: the cytosolic pathway and the vacuolar pathway. The cytosolic pathway, which is the predominant pathway, occurs when exogenous antigens are phagocytosed within the cell endosomes and passed to the cytosol which is then degraded by the proteasome to form antigenic peptides. Digested antigenic peptides are transferred to the ER or phagosomes via TAP transporters and subsequently loaded to MHC class I molecules. Further, peptide–MHC class I bound complexes are later transferred to the cell surface for T cell recognition. The vacuolar pathway, in which the exogenous antigen is digested by proteases present in the lysosomes (mainly by cathepsin S) and transported straight to MHC class I molecules, occurs without any intervention of the cytoplasm [47]. The processes of antigen cross-presentation are described in Figure 1.

### 3.2. Conversing the Significance of Cross-Presentation 

Despite the presence of MHC class I and II presentation as a means of showcasing the antigen to immune regulators, the need to determine the existence of an antigen cross-presentation mechanism poses an interrogatory question, the plausible answers to which are discussed here. The naïve T cells are incapable of migrating to peripheral tissue to encounter cancer cells or viral infection and, additionally, costimulatory molecules are required for stimulating these T cells [48]. However, DCs can migrate into peripheral tissues and sentinel abnormal cells, thereby triggering cross-presentation and causing costimulatory molecules to cast protection against the same [49]. Furthermore, skewing the antigen towards cross-presentation could be a possible immune surveillance mechanism, allowing the antigen to be presented to DCs that may not infect DCs, and also to present the cellular pathogens that never reach the cytosol, so as to alarm naïve T cells to initiate the CTL responses [50]. The cross-presentation could also be a way to circumvent the decline in DC-mediated immune response due to frequently occurring DC death because of viral or bacterial infection. As a result, immunity against such viruses and bacteria that infect DCs would be aided [48]. Furthermore, cross-presentation serves the advantage of prolonged antigen presentation even after the infection is cleared [51,52]. Apart from this, the cross-presentation is able to generate interest due to its probable involvement in the induction of peripheral immune tolerance to self-antigens [48,50].

### 3.3. Comparing Different APCs Based on the Ability to Cross-Present

By far, the most efficacious cross-presentation has been demonstrated to achieve what occurs when an antigen is primed to DCs. The explanation for why DCs mainly perform cross-presentation still remains unknown. However, the factors, such as delayed lysosomal acidification, proteolytic activity, and the ability to maintain a higher pH (low pH favors lysosomal proteases) have been considered to enable cross-presentation. Murine XCR1^+^ DCs express XCR1 (the chemokine receptor), which is most likely present on DCs that cross-present, making them the most dominant APCs [49]. In humans, BDCA^+^XCR1^+^CD141^+^ DCs are considered equivalent to the murine XCR1 [49]. Studies also suggest that Batf3 deficient mice exhibit significant defects in cross-presentation in the absence of XCR1^+^ CD8^+^ DC and CD108^+^ DCs, highlighting the essential role of these DC subsets in cross-presentation [53,54]. Delamarre and colleagues have reported that APCs differ broadly in their lysosomal protease content, which directly reflects in their capacity for antigen degradation in vivo. Macrophages have a high number of lysosomal proteases; therefore, degradation of internalized antigens occurs rapidly, whereas DCs and B cells are poor in lysosomal proteases and, thus, have a limited capacity for degradation. As the DCs degrade the internalized antigens slowly, this results in long-term survival of antigens and longer retention in secondary lymphoid organs. Limited proteolysis also favored antigen presentation. It was speculated that limited degradation of antigens in DCs might allow DCs to cross-present exogenous antigens on MHC I by permitting them to exit from endocytic organelles to the cytosol [55]. 

Although directing antigens to macrophages has been reported to deter cross-presentation due to rapid degradation by lysosomes, CD169^+^ macrophages located in the subcapsular sinus of the lymph nodes have been reported to effectively cross-present large antigens to T cells. Additionally, it has been demonstrated that antigenic peptide-bearing macrophages are just as potent in activating naïve CD8^+^ T cells as DCs in vivo, thereby instigating effector and memory T cell responses [56]. Backer et al. have reported that metallophilic marginal zone macrophages (MMM) work in harmony with spleen DCs to strike CD8^+^ T cell responses [57]. Murine lymphatic endothelial cells have been reported to cross-present under non-inflamed conditions [58]. Many lymph node macrophages can efficiently cross-present the antigens to the T-lymphocytes due to their direct proximity to lymph fluid, which carries many antigens. Liver macrophages are ideal for getting rid of gut-derived antigens by presenting them to the T cells [59]. Since macrophages are more hydrolytic when compared to DCs, their cross-presentation potential can be enhanced either by restricting their acidification or targeting the antigen to the early stage, less hydrolytic endosomes [49]. Apart from these, B cells have been reported to indirectly cross-present antigens derived from keratinocytes, albeit less competency than DCs [60,61,62]. Antibodies derived from B cell form complexes with antigens that become engulfed through Fcγ receptors on DCs. Thus, B cells also participate in cross-presentation [63,64,65,66].

## 4. Biology of Human DCs and Role of DC Subsets in Cross-Presentation 

Human DCs are produced by bone marrow progenitors and are referred to as common myeloid progenitors (CMP). In the presence of a transcription factor, Nur77, CMPs give rise to monocytes, which under inflammatory conditions, further differentiate into monocyte DCs (moDC), while, in the absence of Nur77, CMPs differentiate into common dendritic cell progenitor (CDP), which is further divided into two subsets, namely plasmacytoid DCs (pDC) and conventional DCs (CDC). Langerhans cells (LCs), another kind of DCs, are abundant in the epidermis of the skin [67,68]. The DCs exhibit variety in terms of phenotypes, locations/sources, and immunological functions [69]. The diverse nature of DCs contours the immune response when presented with various pathogens. 

The DCs can communicate with other immune cells in two manners. First, they can communicate through intercellular interactions using specific functional proteins and cell surface markers, such as TLRs [70], lectin-like receptors [71,72,73,74] and, second, they can communicate using soluble factors [70,75,76]. The PAMPs present on the pathogen surface are specific to the PRRs present on the DC surface [77]. These PAMPs are also responsible for the activation and maturation of the immature DCs present in the peripheral tissues, which further generate an immune response against the foreign antigen [78,79,80]. After encountering PRRs on pathogens or subjection to pro-inflammatory cytokines, such as tumor necrosis factor (TNF), interferon γ (IFNγ), or interleukin-1 (IL-1), immature DCs are converted into mature competent DCs bearing high levels of MHCs, and costimulatory and adhesion molecules. The DCs release T cell inviting chemokine. Apart from Ag-MHC-TCR complex-mediated activation, DCs are also activated through other receptors, including B7-CD28 and CD40-CD40L (CD40-ligand). Only the mature DCs cells present in secondary lymphoid tissues, and not immature DCs, can activate T cell response [81].

Various DC subsets reside in human skin, thereby acting as attractive sites to deliver tumor vaccines. The DCs showcase TAAs by processing dying tumor cells and engulfing the live tumor cells [82]. The DCs exhibit various advantageous features to be utilized for triggering anti-tumor immunity, such as DC-mediated cross-presentation, which can stimulate both innate and adaptive immunity. The DCs can effectually engulf antigens and take them to the lymphoid organs. In addition to this, DCs express significant amounts of MHC class I and class II molecules. Furthermore, they express high CD80 and CD86-costimulatory molecules involved in activating antigen-specific T cells. In addition to this, DCs can strike cytokines, such as the IL-12 and IFN-α response [83,84,85]. Furthermore, DC-based cancer vaccines may offer a potential non-toxic, effective, and personalized approach to cancer therapy [83]. Therefore, DCs are being targeted to intensify the immunogenicity vaccines and are regarded as an excellent target for developing cancer immunotherapies. 

The cDCs are professional APCs which are classified into two subsets, cDC1 and cDC2. Here, cDC1 is involved in facilitating the cross-presentation of exogenous antigens to CD8^+^ T lymphocytes. In murine lymphoid tissue, the cDC1 subset is primarily responsible for cross-presenting ovalbumin (OVA) but, in other organs, migratory DCs activate CD8^+^ T cells, making cDC1s a prominent cross-presenting cell type. They are also necessary for the immunotherapy-mediated tumor-specific reactivation of CD8^+^ T cells. Contrary to cDC1, cDC2 does not significantly contribute to antigen cross-presentation in mice, while human cDC2 can be induced to produce substantial amounts of IL-12 and contribute to cross-presentation [86]. The pDCs are multifunctional DCs which can take up exogenous antigens; however, are not able to cross-present them in steady state. However, they can be stimulated by TLRs to cross-present soluble or particulate antigens. While pDCs can serve as antigen-presenting cells, they are substantially less effective than cDCs in this regard [87]. Unlike cDCs, moDCs have not been identified as being capable of transporting antigens to lymph nodes and activating T cells. Consequently, the role of moDCs in eliciting a de novo T cell response is still unclear. However, during inflammatory conditions, moDC recruitment is significantly enhanced [68]. Additionally, it has been shown that moDC, during inflammation, promotes cross-presentation and memory T cell activation [88].

The cDC1 had the highest cross-presentation activity, followed by cDC2 and pDCs, when considering the roles and cross-presentation abilities of the DC subsets, with moDCs being the least likely to cross-present.

### Molecular Basis of Cross-Presentation Efficiency in Steady State DC Subsets

Although all DCs have the potential to cross-present, the kind and state of activation they are in determines whether the immune response will result in cross-presentation or T cell tolerance. The DCs in their steady state are immature and are not fully differentiated to carry out their known functional role as inducers of immunity. However, DCs in their steady state are not inactive and continuously patrol through tissues and into lymphoid organs and catch self-antigens and harmless environmental antigens [89]. Cross-presentation by DCs in their steady state is implicated in both central and peripheral tolerance of CD8^+^ T cells to self and environmental antigens [90]. In the study of Bonifaz et al., OVA chemically conjugated with αDEC-205 antibody was found to be 400 times more efficient in presentation than plain OVA. Targeting of αDEC-205: OVA to the DC receptor DEC-205 in the steady state leads to antigen presentation on MHC I products in a TAP-dependent manner. The OVA was selectively presented by DCs to OT-I, OVA-specific, and MHC class-I restricted T cells, which leads to deletion of T cells; however, αDEC-205: OVA, delivered with a maturation stimulus, leads to T cell expansion, and production of IL-2 and IFN-γ. Thus, DEC-205-mediated processing of OVA for MHC class-I presentation leads to tolerance in the steady state and immunity after DC maturation [91]. Elodie Segura et al., demonstrated that even though the insulin-regulated aminopeptidase (IRAP) and mannose receptor (MR) have a negligible impact on the CD8^+^ T cell priming through steady state DC, their importance for inflammatory DC may be inferred from the fact that moDC deficient in IRAP and MR displayed poor cross-presentation activity, indicating the difference in pathways of steady-state and inflammatory DC [92]. The cDCs and pDCs are the two primary DC populations that can be seen in a steady state [93]. The cDCs are further differentiated into lymphoid tissue-resident DCs and migratory DCs, which have immature and mature phenotypes, respectively [93]. Murine lymphoid tissue cDCs can be further divided into CD8^+^ and CD8^-^ cDCs, which are phenotypically immature in the steady state as opposed to migratory cDCs, which enter the lymph nodes (LN) in a mature condition [94]. Initially, it was discovered that, in the steady state, resident CD8^+^ DCs were more potent at cross-presenting antigens than CD8^-^ DCs, although both DC subtypes were equally effective following receptor-mediated endocytosis [95]. Among murine migratory DCs (CD103^+^ and CD11b^+^), CD103^+^ DCs are the most efficient in cross-presentation [90]. In regard to human DCs, CD141^+^ (CD8^+^ homologue) is the most effective cross-presenting DC for necrotic cell-associated antigens, which may be due to the presence of CLEC9A on the cell surface [96]. The pDC can be found in the blood and LN compartments and are identified by the expression of specific markers, such as CD123 (IL-3R), CD303 (BDCA-2), and CD304 (BDCA-4 or neuropilin-1). Their most prominent characteristic is their capacity to rapidly release significant levels of type I interferons (IFN) in case of viral infection [97]. Due to inadequate expression of the co-stimulatory receptor, low MHC class II levels, and restricted antigen phagocytosis, pDCs are not proficient enough to activate CD4^+^ T cells in a steady state [96]. Owing to the lack of stimulatory signals, pDCs appear to be tolerogenic and are attributed to the induction of T cell anergy and the facilitation of Treg development [98,99]. Furthermore, cDC relies on NOX complexes for successful cross-presentation in the steady state, as they produce ROS, which prevents antigen degradation and facilitates efficient processing and presentation. In contrast to cDCs, pDCs lack the ability to cross-present in a steady state and are independent of the NOX complex; however, their ability to cross-present can be augmented by stimulating TLR7 agonists, which further drives the production of mitochondrial ROS (mROS), amplifying their cross-presentation activity [100].

## 5. C-Type Lectin Receptors in Cross-Presentation

Macrophages, DCs, and B cells possess an inherent property to process internalized antigens for T cell response. Among the APCs, DCs are best and possesses an excellent ability to internalize antigens from microbial and tumor origin for processing and presentation to CD8^+^ T lymphocytes. Myeloid lineage APC-expressed PRRs and CLRs are an important affiliate of these receptors, which bind to PAMPs. The CLRs are known to bind to their ligands in a calcium (Ca^2+^)-dependent manner through their carbohydrate recognition domain (CRD), which serves a range of functions, including antigen uptake and cell–cell interactions [101]. The CLRs are known to bind glycan structures present on pathogens, which makes these CLRs a suitable targeting molecule on the DC cell surface. Endocytosed antigens as a result of the glycan binding to the CLRs, are transported into endocytic compartments where MHC molecules may be loaded. Numerous immune system receptors that perform roles in pathogen identification, glycoprotein turnover, or cell adhesion are members of the CLR family [102]. 

For the targeting of CLRs, monoclonal antibodies were an attractive choice to target CLRs on APCs due to their high affinity and receptor specificity [103]. However, their size prevents them from penetrating the tissue, and the Fc region may also cause nonspecific uptake, Fc-R triggering, or provoke the immune system, which would lead to the removal of the therapeutic antibody. Indeed, this strategy was popularized by Prof. Ralf Steinmann and his colleagues, who targeted the DCs through DEC-205 CLR for the induction of OVA-specific CD4^+^ and CD8^+^ T cell response through an OVA-coupled monoclonal αDEC-205 antibody [104,105]. The success of this strategy opened the way for targeting other CLRs, such as DC-SIGN, langerin, MR, Dectin, CLEC9A, etc., with anti-CLR monoclonal antibodies [106,107,108,109]. In a newer strategy of targeting of CLRs based on their glycan binding profile, glycans were considered for the targeting of CLRs. Glycans were advantageous in targeting, as they are less immunogenic and possess simultaneous CLR-targeting abilities as described in Table 1. The CLRs bind particularly to mannose-, galactose-, or fucose-containing glycan structures found on both self and non-self-protein molecules. However, glycans, unlike antibodies, show less binding affinity, although when presented in a multivalent form, they show high binding affinities to targeted CLRs. Therefore, in recent times, methods for the targeting of CLRs using glycans were considered for improving the outcome of immunotherapy against cancer and infectious diseases. Moreover, harnessing the targeting potential of glycans would lead to the development of specialized “glyco-vaccine” formulations for a desired immune response [110]. 

Antigens targeted to DC-specific receptors elicit powerful T cell-mediated immune responses. To date, antibodies and glycan-based ligands both have been used to target antigens to CRD. Indeed, the strategies to target a specific epitope of the CLR have also been shown to result in enhanced antigen cross-presentation. A study by Tacken et al. showed that targeting CRD and the neck region of CLR DC-SIGN via different antibodies resulted in varying degrees of antigen uptake, processing, and CD8^+^ T-lymphocyte presentation. The OVA which conjugated with anti-CRD antibodies were captured into the late endosomal region, whereas OVA conjugated with anti-neck antibodies showed prolonged retention in early endosomal compartments with 1000-fold increased cross-presentation when compared to unconjugated antigens. Therefore, this study proposed the CLR DC-SIGN neck domain as an interesting target for DC-targeted cancer vaccine approaches [111].

The CLRs are mostly transmembrane receptors, and they exhibit distinctive expression patterns and are capable of binding to a variety of endogenous and/or exogenous antigens leading to various activities [112,113,114]. These receptors are classified into two groups, as follows: Group 1 receptors include MR and DEC-205 and have multiple conserved CRDs in their structures, whereas Group 2 receptors are mostly expressed on DCs and macrophages and have one CRD, which can be the dendritic cell-specific ICAM-3-grabbing nonintegrin (DC-SIGN), langerin, macrophage galactose-type C lectin (MGL), DC immunoreceptor (DCIR), or blood dendritic cell antigen 2 (BDCA-2) receptors. Although Dectin-1 was considered to be a part of the Group 2 receptors, it lacks standard Ca^2+^-dependent CRD, which is present in DC-SIGN [115,116,117]. The CLRs involved in cross-presentation include DEC-205, MR, and DC-SIGN. Furthermore, DEC-205, MR, CLEC9A, and DC-SIGN are examples of CLRs that play a significant part in anti-tumor immunity and can be potential targets for anticancer therapy. The CLRs, such as Dectin-1 and -2, also play an essential role in DC maturation and can generate certain cytokines and chemokines. Indeed, MR, DC-SIGN, DEC-205, and Langerin all generate robust CD4^+^ and CD8^+^ T cell responses; however, Dectin-1 induces a modest CD8^+^ T cell response [118,119,120].

**Table 1:** Comparing antibody-mediated and GNV-mediated CLR-targeting.

	Antibodies Mediated CLR Targeting	GNV-Mediated CLR Targeting	Reference(s)
Delivery to endosome type	Early or late endosome	Early endosome/endo-lysosomal compartments	[111,121,122]
Mode of Ag presentation	Cross-presentation	Cross-presentation	[111,123]
Mode of internalization into the cell	Endocytic receptor-mediated internalization	Internalized into the cell *via* CLR-mediated endocytosis	[2,103]
Type of interactions and affinity	Antibody-mediated interactions are monovalent and of high affinity	Multivalent glycan display for CLR-targeting and low affinity	[124]
Immunogenicity	Anti-CLR antibodies evoke immune response which leads to their elimination	Negligible immune response generated against glycans	[125]

### 5.1. C-Type Lectin Receptors and Their Glycan Preferences 

Glycan-mediated targeting of CLRs on APCs is considered as an attractive strategy for the generation of GNVs, as it can be purified in large quantities from natural sources at a lesser cost. In addition, it provides superiority in terms of binding specificity, flexibility, and spatial orientation, and these properties are beneficial in terms of decorating these glycans on nanocarriers to achieve maximum binding towards CLRs on APCs. Furthermore, CLRs also possess overlapping glycan binding preferences; therefore, they are ideal for the simultaneous targeting of the CLRs. Therefore, careful designing of the nanocarriers so that spacers that attaches the carrier and the glycan are placed in a proper position is essential to achieve efficient targeting [126]. The CLRs have shown wide glycan specificity, and most of the CLRs are expressed on specific subsets of DCs. Regardless of their expression profile, CLRs have the capability to internalize glycosylated antigens [115,117,127,128]. 

The MR displays a binding preference for glycoconjugates terminated with mannose, fucose, or GlcNAc. The MR also showed affinity for sulfated glycans with galactose or GalNAc [129]. The DC-SIGN displays binding affinity for high mannose- and fucose-containing glycan structures. The DC-SIGN binds to Man_9_GlcNAc_2_-Asn-containing glycopeptides, and binding decreases with smaller mannose-containing glycans [130]. Fucosylated glycan binding was also found with DC-SIGN in the binding order of Le^b^ > Le^y^ > Le^a^ [131]. Langerin binds mannose, fucose, and GlcNAc facilitated by oligomerization in the neck region [132]. Additional binding of langerin with sulfated Le^x^-glycans was also observed; however, high mannose glycans reported weak binding. Furthermore, MGL has shown a binding preference for glycoproteins or glycosphingolipids containing α- or *β*-linked GalNAc as terminal residues. Additionally, MGL binding with Tn antigen (α-GalNAc residue) was also reported [133]. Glycan specificity of DCIR was reported towards mannotriose, Le antigens (Le^a^, Le^b^), and the sulfo-Le antigen (sulfo-Le^a^) [134]. The BDCA-2 possess two separate kinds of binding sites, namely primary and secondary, for mannose- and galactose-containing glycans, respectively [135]. Dectin-1 binds with *β*-glucans with *β*-1,3 and/or *β*-1,6-linked glucans [136,137]. Therefore, the wide array of glycan recognition by CLRs provides an opportunity to target CLRs on APCs for immunotherapeutic purposes and they are summarized in Table 2.

### 5.2. Toll-Like Receptors (TLRs), Adjuvant Activity and Their Role in Enhancing Cross-Presentation

The TLRs are vital contributors in boosting the effectiveness of GNVs. They are crucial for antigen uptake as, upon recognizing PAMPs, they induce DC maturation, which in turn aims to facilitate enhanced antigen presentation [138]. In steady-state conditions, antigen recognition by the CLRs induces immune tolerance; however, in inflammatory conditions, the CLR binding to antigens in the presence of TLR triggering induces DC maturation and further immune activation [73]. Using an antigen-conjugated TLR agonist has been shown to be an effective strategy for improving antigen uptake, presentation, and T cell priming [139,140]. Therefore, targeting both CLRs and TLRs is necessary to achieve a desired immune response [141,142]. A range of different TLR-agonists, including poly I:C (TLR3 agonist), LPS, and monophosphoryl lipid A (MPLA) (TLR4 agonist), imidazoquinoline compounds (TLR 7/8 agonist), and CpG (TLR9), when encapsulated in the NP-based antigen delivery system, enhanced DC maturation and antigen presentation [143,144,145].

**Table 2:** Comparing various CLRs present on DCs, their expression pattern, ligand specificity, involvement in cross-presentation of antigens, and immunological (humoral and cellular) responses.

CLRs and Their Synonyms	Expression/DC Subtype	Ligands	Cross-Presentation Activity	Humoral and Cellular Response	References
DEC-205/ CD205	Expressed by thymic epithelial cells, subsets of DCs (peripheral DCs, splenic/lymph node DCs, dermal/interstitial DCs, and LCs); homologous to MR family	Apoptotic and necrotic cell-derived antigens, CpG oligonucleotides	Effective cross-presentation of tumor- or pathogen-derived antigens	Induce efficient cellular (CD4^+^ and CD8^+^ T cell) and humoral responses; however, DCs activation by adjuvants required	[119,146,147,148]
Dectin-1/CD369	Expressed by human monocytes, macrophages, DCs; mouse cDC2	*β*-glucans (with *β*-1,3 and/or *β*-1,6-linked glucans)	Uptake and cross-present cellular antigens	Strong CD4^+^ T cell response but weak CD8^+^ T cell response	[149,150,151,152,153,154]
DC-SIGN/ CD209	Expressed by moDCs and dermal CD14^+^ DCs	High-mannose- and Fucose-containing glycans, Lewis antigens	Antigen targeting to DC-SIGN leads to cross-presentation	Strong CD4^+^ and CD8^+^ T cell response	[125,155,156,157,158]
Langerin/CD207	Highly expressed by LCs, dermal DCs in both mice and humans	Mannose, fucose, N-Acetylglucosamine (GlcNAc), *β*-glucans	Langerin mediated cross-presentation in LCs	Induce humoral response and CD8^+^ T cell activation	[159,160,161,162,163]
MR/ CD206	Macrophages, human moDCs, mouse BMDCs	Glycoconjugates terminated with mannose, fucose, or GlcNAc. Affinity towards sulfated glycans is also present	MR-mediated targeting of antigens, directs antigens to early endosomes and leads to cross-presentation	Targeting MR elicit strong cellular and humoral immune response	[164,165,166,167]
MGL/ CD301	DCs, macrophages, dDCs, murine pDC	Terminal GalNAc, Tn antigen (α–GalNAc), glycan antigen LDN, sialyl-Tn	MGL1 mediates TLR signaling independent of cross-presentation	MGL2 targeting induces Th2 skewed humoral response, Th1 skewing of CD4^+^ T cells and enhanced CD8^+^ T cell priming by glycan-modified antigen targeting	[133,168,169,170]
DCIR/ CD367	DCs, monocytes, neutrophils, B cells and activated T cells	Mannotriose, Le^a^, Le^b^, and Sulfo- Le^a^	DCIR-mediated antigen targeting leads to cross-presentation	DCIR targeting induces CD8^+^ T cell response	[134,171]

Le refers to lewis antigens where Le^a^ is Galβ1-3(Fucα1-4)GlcNAc and Le^b^ is Fucα1-2Galβ1-3(Fucα1-4)GlcNAc.

## 6. DC-Based Immunotherapies versus DC-Targeted Cross-Presentation

In humans, autologous DCs have been used to augment the quality and magnitude of tumor-specific immune responses. DC-mediated immunotherapies can be broadly classified into two groups, namely in vivo DC-targeted vaccines and ex vivo antigen-loaded DC-based vaccines [27]. However, the challenges associated with DC immunotherapies, such as the essential prerequisite, examining the formulations for plausible contaminants and alterations in DCs owing to in vitro dealings, increase the production costs, making it highly expensive. Hence, targeting DCs without having to remove them from the body is an absolute necessity and may possibly be more effective and cost-effective. On the other hand, the ex vivo process involves the isolation of DC-progenitors from the patient’s blood or bone marrow, expansion of DCs from these progenitors, and subjecting DCs to tumor antigens followed by reinsertion of these cells into the patient’s body. Such vaccines have been demonstrated to be effective in treating prostate cancer, malignant glioma tumors, renal cancer, and hepatocellular carcinoma [172]. Studies have also shown that TAA-based personalized DC vaccines in which DCs were transfected with mRNAs of overexpressed TAA from the patient can be used to treat solid tumors, such as glioblastoma multiforme and advanced lung cancer, as the patients transfected with TAA-based DC vaccines developed antigen-specific CD4^+^ and CD8^+^ T cells [173]. Although this approach has promising attributes, at present, it is unlikely to be put into practice due to the prerequisite that it must be tailor-made and standardized for each individual and, hence, is expensive, laborious, and time-consuming, and requires expertise to perform the intricate procedures. In addition to this, the DCs may undergo alterations during in vivo induction and may trigger an immune response on reinfusion [27]. 

## 7. Orienting Antigen towards Cross-Presentation

The generation of anti-tumor responses depends on the stimulation of IFN-producing CD4^+^ T cells as well as the cross-presentation of TAAs, which further enhances the CD8^+^ effector T cells. The antigen cross-presentation occurs under certain specific circumstances and, thus, steering the fate of an antigen towards cross-presentation is of prime importance. The type of DC receptor being targeted is of crucial importance as it decides the fate of the tumor antigen and further elects the intracellular pathway of antigen routing [174]. The intracellular trafficking factors, such as the regulation of antigen degradation, export to the cytosol, ER recruitment, and MHC- class I: neo synthesis or recycling, etc., have been reviewed by Alloatti and colleagues [175]. The process by which an antigen is internalized is also crucial as it determines whether and how an antigen will be cross-presented. A classical study conducted by Li et al. reported that cell-associated OVA cross-presents with much higher efficiency than in its soluble form in vivo [176]. Antigens internalized via fluid phase pinocytosis are inefficiently cross-presented, as a very high concentration of antigen is required for a peptide–MHC I complex. This is why soluble antigen fails to prime CD8^+^ T cell responses [177,178,179]. However, the particulate forms of antigens internalized via phagocytosis or macropinocytosis are cross-presented efficiently and produce more potent T cell responses than soluble antigens [177,178,180]. Burgdorf and colleagues have shown that OVA internalized through pinocytosis routes to CD4^+^ T cells, whereas when captured by DC through MR-mediated endocytosis, it enters the cross-presentation pathway in the presence of a TLR4 co-signal [181,182]. Additionally, the CTL response generally has high avidity and leads to action of granzymes and perforins against cancer, which have been demonstrated to confine tumors; as a result it may help increase the patient’s lifespan [183,184].

## 8. Using Nanovehicles for Tumor Antigen Cross-Presentation

Several advantages of nanocarriers as cancer therapeutics have been extensively reviewed in various reports to illustrate the increased therapeutic efficacy of the drugs. Shielding when in circulation, target specificity, better accumulations, and controlled release inside tumors are some of the advantages of nanocarriers [11,185,186,187,188,189]. Nanovaccines, usually comprising of tumor antigens along with apt immune stimulators, have been utilized to develop immunotherapy against cancer wherein the tumor antigen was either presented to stimulate direct T cell response or indirectly to DCs. Such nanovaccines are built on the underlying principle involved in T cell and/or DC-mediated immune response [174]. Encapsulating antigens in a nanocapsule provides a protective shelf against the endosomal proteases and has been shown to enhance the sustenance of antigens in endosomes, thus, instigating prolonged release of the antigen and, by this means, improving the chances of cross-presentation [2,190,191]. For obtaining optimal immune response, TAAs are usually administered along with an appropriate adjuvant. 

### 8.1. Glycan-Conjugated Nanovaccines Targeting DCs

**Figure 2:**
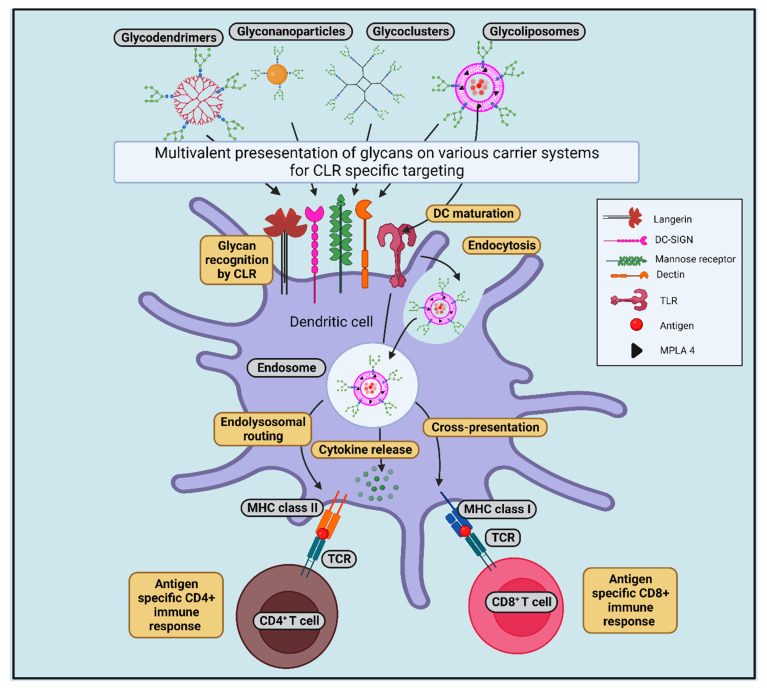
Multivalent presentation of glycans on various carrier systems for enhancing the antigen presentation to achieve effective T cell response. Glycan-modified nanocarriers present glycans in a multivalent form. Additionally, TLR ligands can also be incorporated with these glyconanocarriers. Glyconanocarriers, such as (i) glycoliposomes, which can be used for encapsulation of whole tumor antigens and adjuvants, (ii) glycodendrimers, which can be prepared with the desired number of glycans and peptides, and (iii) synthetic glycoclusters, which can also be prepared with tumor antigenic peptides. These glycan-modified nanocarrier systems loaded with tumor antigens are effectively internalized by DCs in a CLR-specific manner. The internalized antigens are presented by the MHC class I and II pathway for CD8^+^ and CD4^+^ T cell responses, respectively. The addition of MPLA, a ligand of TLR4 in glycoliposomes targeting CLR DC-SIGN, enhances DC maturation and cross-presentation of tumor antigens to CD8^+^ T cells (Created with BioRender.com accessed on 27 September 2022).

Glycans are complex carbohydrates that are present on mammalian cell surfaces. Glycans play an essential role in various cellular mechanisms, such as cell adhesion, signal transduction, molecular trafficking, endocytosis, etc. The immunogenicity of glycans is below par, as they are expressed throughout the body [37]. The direct encounter of malignant cells bearing TAAs to CTLs elicits an inadequate cytotoxic immune response [192], whereas the cross-presentation leads to heightened, sustaining, and a comparatively more effectual immune response. The DCs can cross-present not only the antigens from apoptotic cells but also the antigens encapsulated in NPs, which are then destined to specific uptake receptors expressed by DCs. Polymer particle-based vaccine delivery systems have been used widely for antigen delivery to DCs for vaccine development; however, these biodegradable polymers do not have PAMPs and, therefore, their interaction with DCs is limited. Furthermore, certain immune receptors of DCs have been demonstrated to be immune suppressed; hence, targeting antigens to DCs does not ensure an effective immune response [193]. Glycans are involved in cellular communication between immune cells mediated through CLRs. The CLRs recognize and bind to specific glycans, uptake the molecule, and can thereby orchestrate immune responses. Therefore, developing glycan-based nanomedicines can provide future directions to facilitate DC-based strong immune responses so as to destroy cancer cells as shown in Figure 2. The PRRs present on the DCs are specialized cell surface markers that identify ligands, which are antigenic components present on microbes. These ligands, on binding to the PRRs, stimulate T cell immunity. Various PRRs and their respective ligands have been identified. However, only two main PRRs-CLRs and TLRs are known to be involved in the antigen recognition [27], and can be exploited for formulating vaccines which aim to stimulate DC-mediated immunity. To improve the quality of the immune response in terms of amplitude, sustainability, dosage amount, and frequency of immunizations, a number of factors must be taken into consideration. However, such immune response could be inadequate to effectively target and eliminate the antigens. The ability to recognize glycans by DCs can help us invigorate the DC-based translational strategies against cancer [2,119,131]. 

### 8.2. Targeting CLRs by Glycans (in the Form of Glycosylated Antigens/Glycan-Modified Nanocarrier) to Induce Cross-Presentation 

Targeting of CLRs via glycan-modified antigens mimics natural CLR–ligand interaction as it provides information about CLR-mediated signaling and antigen routing subsequent to glycosylated antigen binding under physiological settings [110]. In recent times, targeting of DC-SIGN via glycan-modified antigens, liposomes, and dendrimers results in a robust CD8^+^ T cell response, confirming the diversion of antigens towards cross-presentation pathway. 

In a study by Singh et al., it was reported that modification of OVA antigens with Le^x^ or Le^b^ for targeting of the DC-SIGN on transgenic DCs induces both arms of cellular immune response (CD4^+^ and CD8^+^ T cell responses) and, in comparison to plain OVA, modified OVA was 10-fold more cross-presented [194]. In another study by Streng-Ouwehand and colleagues, it was demonstrated that Le^x^ alteration of OVA guided OVA to MGL1, promoting Th1 skewing of CD4^+^ T cells and elevating cross-priming of CD8^+^ T cells. Cross-presentation of Le^X^-conjugated OVA antigens was found to be MGL1-dependent, and reduces both the high antigen dose requirement and TLR dependence [170]. Conjugation of liposomes with Le^b^ and Le^a^ for DC-SIGN targeting enhances both binding and DC-SIGN-mediated internalization of these liposomes by bone marrow-derived DCs (BMDCs) and have also shown efficient CD4^+^ and CD8^+^ T lymphocyte responses against melanoma antigen, MART-1 [158]. When TLR4 ligand MPLA was incorporated as an adjuvant into Le^X^-conjugated liposomes, these glycoliposomes were uptaken by human DCs in a DC-SIGN-dependent manner, and inclusion of MPLA resulted in DC maturation and proinflammatory cytokine release associated with increased cross-presentation of the glycoprotein (gp) 100 _(280–288)_ peptide melanoma antigen to CD8^+^ T cells [195]. As human skin contains various DC subsets, therefore, it is considered an attractive strategic point for anti-tumor vaccine delivery. When the skin explant model was used for intradermal delivery of the melanoma-associated gp100_280–288_ peptide and MPLA-containing liposomes, these liposomes were efficiently taken up by CD14^+^ dermal DCs and MPLA inclusion resulted in a significant increase in CD8^+^ T cell response [196]. Interestingly, Fehres et al. demonstrated different degrees of size requirement for DC-SIGN- and langerin-mediated uptake and antigen presentation by DCs and LCs. Since CLR DC-SIGN and Langerin both recognize the difucosylated oligosaccharide Le^y^, Le^y-^modified liposomes were prepared. The Le^y^-modified liposomes bound to DCs and endocytosed by DC-SIGN^+^ DCs; they showed efficient antigen presentation to both arms of the cellular immune response (CD4^+^ and CD8^+^ T cells); however, the same liposomes were bound to LCs but failed to be endocytosed, and no enhanced antigen presentation was observed. Surprisingly, Le^y^-modified long synthetic peptides targeting LCs via langerin resulted in an enhanced antigen cross-presentation; however, DCs bound to Le^y^-modified long synthetic peptides via DC-SIGN failed to become internalized or to cross-present the antigen [197]. Similarly, Garcia-Vallejo et al. showed that generation three dendrimers with 32 Le^b^ glycan units having similar chemical structures and enhanced ligand density are sufficient to increase binding to DC-SIGN, internalized by moDCs, and cause further routing of these dendrimers to lysosomal compartments for enhanced CD4^+^ and CD8^+^ T cell responses. Therefore, this study confirmed that multivalency is a crucial factor in this enhancement [198]. 

Targeting of MR via monovalent or multivalent mannosides conjugated to lipopeptides, antigens, and nanocarriers also resulted in enhanced uptake via MR as well as cross-presentation of antigens [199]. Mannosylation of long tandem repeat peptides has shown enhanced uptake of divalent mannosylated MUC1 by MR-positive macrophages and DCs [200]. In another study, mannosylation of synthetic long peptides also showed enhanced internalization via MR in murine APCs and further targeting to early endosomes and cross-presentation, which leads to CD8^+^ T cell activation [201]. Apostolopoulos and colleagues, in their studies, showed that oxidized mannan linked to MUC1 (oxi-mannan-MUC1) binds to MR, becoming internalized and very efficiently presented by MHC class I molecules (1000 times more) in comparison to their reduced-MUC1 form [202]. Ex vivo targeting of MR-bearing macrophages with oxi-mannan-MUC1 followed by adoptive transfer, which is efficient in presenting MUC1 to T cells, results in high-frequency CTL generation and protection against a tumor challenge [203]. An immunotherapeutic study in breast cancer patients (stage II) with oxi-mannan-MUC1 up to 15-year clinical follow-up showed reduced recurrence rate [166]. 

Another strategy for transferring antigens to the cytosol for cross-presentation is studied using pH-sensitive liposomes [122,204,205,206,207]. Glycan-based pH-sensitive liposomes were prepared for transferring the antigens to the cytosol and inducing APC maturation via glycan interaction. Curdlan and α-mannan both possess carboxylated moieties that induce the maturation of DC cell lines better than the dextran derivatives. This observed maturation of DC lines was thought to depend upon the recognition of curdlan and mannan by CLR Dectin-1 and -2, respectively [206]. Furthermore, curdlan derivative-modified liposomes transferred model antigens to the cytosol of DC, which induced antigen-specific cellular responses and regression of tumors [207]. Modification of curdlan derivatives with mannose residues induces enhanced attachment of liposomes with macrophages and further cross-presentation [122]. Hyaluronic acid (HA) and chondroitin sulfate (CS)-based pH-sensitive glycans were also prepared, and these derivative-modified liposomes successfully accomplished antigen release into the cytosol of APCs through pH-responsive mechanisms and CD44-dependent cellular association, eliciting the antigen-specific cellular arm of the immune response [204,205]. The anti-tumor activity of curdlan-based pH-sensitive glycan-modified liposomes was found to be higher than the HA- or CS-based pH-sensitive glycan-modified liposomes. Therefore, these studies highlighted the importance of glycans in achieving high adjuvant activity, the release of antigen in the cytosol, and further cross-presentation. More importantly, a precise design of CLR-targeting glycans is an absolute necessity for the design of nanovaccine formulations. 

**Figure 3:**
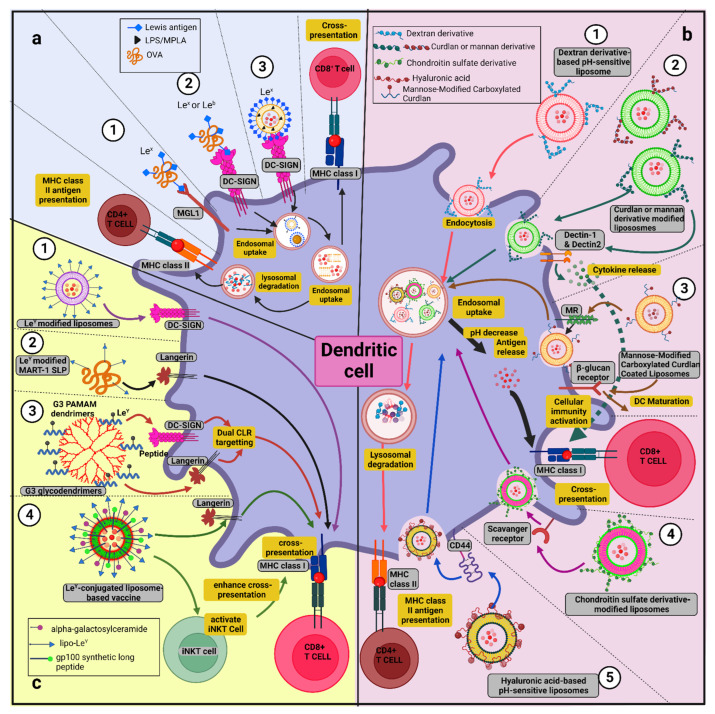
Glycan-mediated strategies for DC targeting via CLRs for enhanced antigen cross-presentation. (**a**) Lewis antigen-modified ovalbumin (OVA)/nanocarriers are as follows: (1) Le^x^-modified OVA redirected to MGL1 promotes Th1 skewing of CD4^+^ T cells and CD8^+^ T cell cross-priming; (2) Le^x^- or Le^b^-modified OVA antigens are internalized by DC through DC-SIGN-mediated uptake which promotes CD4^+^ and CD8^+^ T cell responses; (3) the MPLA-modified Le^x^ conjugated liposomes are internalized by a DC-SIGN specific manner into the DCs to further enhance CD8^+^ cross-presentation; (**b**) Glycan-coated pH-sensitive liposomes are as follows: (1) carboxylated dextran derivative-modified pH-sensitive liposomes enhance the pH-sensitive endo-lysosomal degradation and promote CD4^+^ and CD8^+^ immune responses; (2) Curdlan and mannan derivative-modified liposomes are recognized by Dectin-1 and -2, respectively, which are endocytosed into the DCs and, due to weak pH conditions, the endosome disrupts to release antigens into the cytosol for proteasomal degradation to be further presented on the cell surface, which leads to enhanced CD8^+^ T cell response; (3) Mannose-modified curdlan-coated liposomes are recognized by MR which transfers the liposome to the endocytic compartment for degradation and, furthermore, the pH-sensitive environment induces the antigen release into the cytosol for proteasomal degradation for further antigen presentation to CD8^+^ T cells. Additionally, they are also recognized by *β*-Glucan receptors to promote DC maturation; (4) To induce antigen-specific cellular immunity, chondroitin sulphate derivative-modified liposomes would be specifically taken up by APCs cells via scavenger receptors and these encourage cytokine production from the cells as well as the endosomal escape of antigenic proteins through pH-responsive membrane destabilization; (5) Hyaluronic acid-based pH-sensitive polymers are recognized by CD44 present on APCs. These liposomes were successful in delivering model antigenic proteins into the cytosol of DCs and releasing the degraded antigenic peptide into the cytosol, which was then loaded onto the MHC class I molecules and elicited CD8^+^ recognition; (**c**) Glycan-coated nanocarriers targeted to LCs and dermal DC are as follows: (1) Le^y^-modified liposomes when presented to the dermal DC were taken up via DC-SIGN which leads to enhanced CD8^+^ cross-presentation; (2) Le^y^-modified MART-1 synthetic long peptides are taken up through langerin in the LCs to promote cross-presentation; (3) The G3 glycodendrimers induce dual targeting of langerins and DC-SIGN which promotes antigen cross-presentation; (4) Liposomal vaccine containing synthetic long peptides and alpha-galactosylceramide (α-GC) conjugated with Le^y^ antigen, which promote CD8^+^ T cell response and induce iNKT cells activation, which enhances cross-presentation (Created with BioRender.com accessed on 27 September 2022).

The above mentioned CLR targeting strategies are briefly showed in Figure 3. 

## 9. Parameters to Be Considered for Developing GNVs

The ideal GNV should be able to deliver cancer antigens to DCs effectively, stimulate cross-presentation, should lead to increased CTL response, and should overcome the immune modulatory signals of tumor. To achieve all these objectives, the knowledge of factors, such as sizes, geometries, and physical properties of pathogens contributing to host–pathogen interactions may help ground the rational design of the GNVs. The type of NP (see Table 3) and particle size govern the fate of the antigen by selecting the suitable antigen processing pathway in DCs. The choice of DC receptor used for targeting is crucial to better target the antigen to the DCs. While coating the NP with ligand, the polysaccharide composition, glycan density, spacing and spatial orientation, and the degree of mobility of the ligand need to be taken into consideration as these play an important role in its interaction with the DC receptor [208,209]. Furthermore, the cell surface carbohydrate–protein interactions are usually multivalent, meaning that multiple carbohydrates interact with the same protein. In the case of NP-mediated immune response, a multivalent carbohydrate display has been demonstrated to be more effective. A geometrically complementary ligand that can fit the DC’s cell surface receptor can be synthesized. Coating NPs with multivalent synthetic analogs that can mimic the cell surface carbohydrates may provide a potential solution for effective DC-targeting and cross-presentation. The DC-SIGN and DC-SIGNR receptors are found to bind to high mannose-binding oligosaccharides, among which the Man_9_GlcNAc_2_ polypeptide has been demonstrated to exhibit the highest affinity [130]. Moreover, the overall topology of Man_9_GlcNAc_2_ is better defined due to high linkage flexibility. Other ligands with similar or better cross-presentation responses need to be identified. Recent researchers are also taking into account the glycomimetics, such as fluorinated glycomimetic, to study whether they have better affinity to the DC receptors than the natural molecule [210]. The choice of antigen—between mutated or shared self-antigen—is a critical factor to be considered [211]. Tumor-associated carbohydrate antigens (TACAs) can be regarded as poor, intermediate, or strong immune response-generating antigens. The choice of strong TACAs helps render a strong immune response, thereby helping us to eliminate cancer. Soluble antigens are known to be translocated into the cytosol and cross-presented, but with poorer efficiency than particulate matter. The activation of DCs, which is achieved with immune stimulators, is equally important to achieve successful cross-presentation [212]. In order to avoid long-term toxicity, the biodegradability and clearance of the nanoparticles following antigen or drug delivery must also be taken into consideration [213]. 

### 9.1. Types and Fabrication Strategies of GNVs and Their Associated Benefits in Anti-Tumor Immunotherapy 

Various strategies for the fabrication of GNVs are employed in the diversion of antigens towards cross-presentation pathways. Fabrication of glycan-conjugated/modified liposomes (glycoliposomes), glycan-modified pH-sensitive liposomes, dendrimer (glycodendrimer), and PLGA nanoparticles are reported in the targeting of CLRs, maturation of APCs, and in enhanced CD8^+^ T cell response [221,222]. Fabrication strategies and benefits of various GNVs in cancer immunotherapy are described below and summarized in Table 4.

#### 9.1.1. Glycoliposomes

Lipid-based nanocarrier systems are an excellent choice for antigen delivery to DCs as they are naturally derived and have the advantage of biocompatibility, as well as the possibility of tailoring the adjuvant effects. Liposomes (phospholipid-based membrane vesicles) are an excellent choice for the delivery of the antigens, as they are safe and well tolerated by the immune system. In addition, liposomes can be diverse in their size and lipid composition, thereby affecting the adjuvant properties of the prepared liposome. Liposomes are known to effectively co-deliver adjuvants along with antigens by mimicking the pathogen encounter by DCs, thus, enhancing the chance of eliciting cross-presentation by DCs [223]. Hence, standardization of the particle size and composition are of pivotal importance. Surface charge modification of the liposomes can be performed to promote enhanced uptake by DCs and, due to their vesicular nature, membrane-associated and soluble antigens can be encapsulated in the liposomes [2,221,224,225,226].

In a series of studies, Van Kooyk and colleagues prepared glycoliposomes for the targeting of CLRs for enhanced CD8^+^ T cell response. Glycan-modified PEGylated (prepared from a mixture of egg phosphatidyl choline (EPC-35): 1,2-distearoyl-sn-glycero-3-phosphoethanolamine-N-[maleimide (polyethylene glycol)-2000](PEG-mal): PEG-DSPE): cholesterol (Chol)) and non-PEGylated (prepared from a mixture of EPC-35: Ethanolamine Phosphoglyceride (EPG): 1,2-dioleoyl-sn-glycero -3-phosphoethanolamine-N-[4-(p-maleimidophenyl) butyramide] sodium salt (MPB-PE): Chol) liposomes encapsulating model antigen OVA were synthesized using a phospholipid mixture and film extrusion method. Here, 1,1′-dioctadecyl-3,3,3′,3′- tetramethylindodicarbocyanine, 4-chlorobenzenesulfonate salt (DiD) was included as a fluorescent marker in the lipid bilayer. Liposomes were coupled with Le^x^ and Le^b^ glycans via a maleimide group. Only glycan-modified non-PEGylated liposomes were able to bind to DC-SIGN expressed by BMDCs generated from DC-SIGN transgenic mice, and no binding was observed with glycan-modified PEGylated liposomes. It was anticipated that PEG sterically hindered the interaction between glycan-modified PEGylated liposomes with DC-SIGN and, therefore, PEG should be avoided in CLR-targeted liposome preparation [227]. Adopting a similar strategy, OVA/MART-1 peptides containing liposomes were prepared and glycan-coupling to these liposomes was carried out with thio-activated Le^x^ and Le^b^ glycans (glycan derivatives with a thiol group were prepared through reducing end’s reductive amination with cysteamine) via a thiol–ene reaction with maleimide groups of MBP. Glycan modification of liposomes resulted in enhanced binding and internalization by human DC-SIGN expressing BMDCs. Furthermore, due to the addition of LPS, 100-fold efficient antigen presentation was found. Under a similar setting, MART-1 peptides containing glycoliposomes showed efficient antigen presentation to MART-1 specific CD8^+^ T cell clones [158]. To study the effect of the incorporation of various TLR ligands in glycan-modified liposomes, EPC-35: EPG-Na: Chol (in a molar ratio of 3.8:1:2.5) phospholipids were combined with TLR ligands (MPLA (2 mol%), Pam3CysSK4 (1 mol%), or R484 (4 mol%)). Poly I:C, a hydrophilic TLR ligand, and antigenic peptide gp100_280–288_ were encapsulated during the hydration step. Furthermore, Le^x^-glycolipid was inserted into these liposomes. After 15 min of vigorous stirring and storage overnight at 4 °C, the liposomes suspension was collected and resuspended in the same buffer. Inclusion of the TLR4 ligand MPLA in glycoliposomes significantly enhanced antigen cross-presentation of the gp100 antigen [195]. 

Further improvement in anti-tumor immunotherapy was achieved when palmitoylated antigen and lipo-Le^Y^, together with alpha-galactosylceramide (αGC), were combined in a single liposome for their in situ delivery to the skin APCs which enhanced antigen presentation to CD8^+^ T cell and invariant natural killer T cell (iNKT) activation. To achieve this, EPC-35: EPG-Na: Chol (in a molar ratio of 3.8:1:2.5) were added to a solution of chloroform/methanol. Lipophilic fluorescent tracer DiD (0.1% in mol), palmitoyl-gp100/MART-1 (400 µg), lipo-Le^Y^ (1.5 mg), and NKT cell activator αGC (30 µg) were added in the mixture. The obtained lipid film was hydrated in a HEPES buffer of pH 7.5, and liposomes were sized by extrusion. Antigen and lipo-Le^Y^ that had not been encapsulated were then removed using ultracentrifugation [228]. Inclusion of ganglioside as the targeting ligand for human CD169^+^/SIGLEC-1^+^ APCs and TLR4 ligand MPLA in liposomes also resulted in cytokine production, robust tumor antigen cross-presentation, and tumor antigen-specific CD8^+^ T cell response activation, which confirmed another nanovaccine platform for enhancing anti-tumor immunotherapy [229]. Incorporation of inflammasome stimuli 1-palmitoyl -2-glutaryl-sn-glycero-3 -phosphocholine (PGPC)/muramyl dipeptide in these liposomes did not further enhance its potency [230].

#### 9.1.2. pH-Sensitive Glycan-Modified Liposomes

Cytosolic delivery of antigens is considered as a major pathway in antigen cross-presentation. The pH-sensitive liposomes have the capability to release their inner material in response to a change in pH, as well as destabilization of the endosomal membrane to release the model/tumor antigen to the cytosol. Since these liposomes are created through surface modification using pH-sensitive components, they are also known as stimuli-responsive liposomes. Viral fusogenic proteins tagging to liposomes are also used in promoting antigen release into the cytosol [222,225]. Glycan-modified pH-sensitive polymers were designed to target APCs via recognition of CLRs, to induce maturation of APCs as well as to promote release of antigen into cytosol. Various poly (carboxylic acid)-based pH-sensitive polymers were synthesized and used in the preparation of pH-sensitive liposomes [207]. The pH-sensitive moieties were linked to polysaccharide dextrans by reacting with 3-methylglutaric anhydride, and the resulting 3-Methyl-glutarylated dextran (MGlu–Dex) was used in the preparation of pH-sensitive liposomes [206]. Similarly, 3-methylglutarylated (MGlu) groups were also introduced to curdlan and mannan to synthesize carboxylated curdlan (MGlu–Curd) and mannan derivatives (MGlu–Man). Liposomes and polymer-modified liposomes were prepared by using the following strategy. Briefly, to a thin and dry membrane of egg yolk phosphatidylcholine (EYPC), 1 mL of OVA 4 mg/mL in PBS pH 7.4 was added, followed by vortexing the mixture at 4 °C and further hydration by freeze-thaw and sizing by extruding through a polycarbonate membrane (100 nm size). Free OVA was removed from OVA-loaded liposomes using a Sepharose 4B column. Polymer-modified liposomes were prepared with a similar procedure using dry membrane of a lipid mixture with polymers (lipids/polymer = 7/3 *w*/*w* ratio). Then, MPLA (4 g/mol lipids) was introduced in both the liposomes for the induction of an immune response. Incorporation of carboxylated curdlan and mannan into the liposomes gave benefits in terms of maturation of DC, targeting to Dectin-1 and -2 on APCs, delivery of antigens to the cytosol of DCs, and antigen-specific cellular immune response. Curdlan derivatives with MGlu units (as a pH-sensitive unit) and decylamidated units as an anchor unit to liposomal membrane (MGlu–Curd-A) were further functionalized with mannose. Mannose-functionalized curdlan derivatives which were modified onto antigen-loaded liposomes showed superior pH-sensitivity compared to the original curdlan derivatives [122]. The CS and HA reaction with 1,2-cyclohexanedicarboxylic acid anhydrides gave carboxylated derivatives and CS and HA derivatives. Liposomes prepared with CS- and HA-modified polymers were used in selective targeting of CD44 on APCs, cytosolic delivery of antigens, and induction of cellular immune response [204,205]. The β-glucan-based pH-sensitive polysaccharides have been proven to be efficient in cytosolic antigen delivery and adjuvant activity. However, adding cationic lipids further enhances their adjuvanticity and the cellular interaction with DCs resulted in increased cytokine production from DCs, as well as the induction of a robust immune response [231].

#### 9.1.3. Glycodendrimer

Multivalent presentation of glycans is necessary for optimum targeting of CLRs, CLR-mediated internalization, and lysosomal delivery, followed by Ag-specific T cell proliferation and cytokine secretion. Multivalent platforms, such as poly(amido amine) PAMAM dendrimers, are excellent for antigen targeting. Dendrimers give a range of advantages as they are repetitively branched, compact, and have a synthetic molecular structure with functional groups for the conjugation of glycans and antigens by simple chemical reactions [232]. Their compactness, flexibility, and solubility provides an opportunity for defined geometric orientations, and glycans, as well as dendrimers, can be engineered with the required amounts of glycan and peptide antigens. 

A glyco-dendrimer-based anti-tumor vaccine with dual CLR (DC-SIGN, Langerin) targeting was prepared for the targeting of multiple skin DC subsets for improved anti-tumor therapy. Branched PAMAM dendrimers were used as a scaffold for melanoma specific gp100 synthetic long peptides and a common ligand Le^Y^ (for DC-SIGN and Langerin) for preparing the multivalent glyco-dendrimer. In brief, glyco-dendrimers were prepared through thiol–ene-mediated reactions. The PAMAM dendrimers with G0 and G3 generations were functionalized with maleimide or LC-SMCC bifunctional cross-linker. Dendrimers were then purified and loaded with gp100 peptides through its C-terminal cysteine. Excess peptides were removed and labeling and glycation was achieved by unmasking N-terminal thioproline and reacting it with AF488/Lewis^Y^ pentasaccharide maleimide. Simultaneous targeting of multiple human skin DCs through DC-SIGN and langerin in combination with TLR stimulation enhanced cross-presentation and gp100-specific CD8^+^ T cell activation [233].

#### 9.1.4. Glyconanoparticles

Xu and colleagues have designed mannan-decorated pathogen-like polymeric nanoparticles (MPVax) as a protein vaccine carrier for inducing robust anti-tumor immune response. Synthesis of polylactic acid (PLA) was carried out using a ring opening polymerization of lactide with stannous octate as a catalyst. Here, PLA and polyethylenimine (PEI) were conjugated together to result in PLA-PEI. Then, 2g PLA (0.133 mmol) was dissolved into DMSO, followed by the addition of 1.2eq carbonyldiimidazole, and the solution was further stirred for 12 h before DMSO-dissolved PEI was added, and the reaction was proceeded for another 24 h. After this, the final product was recovered after dialysis against water and lyophilized. The PLA-PEI inner core (PVax) was formed through nanoprecipitation, in which the PLA-PEI was dissolved in 1 mL of DMSO, and this solution was added to 15 mL 10 mM HEPES buffer with sonication followed by dialysis and volume adjustment to 20 mL before it was stored at 4 °C. For MPVax containing CpG/OVA, OVA and CpG was added to PVax solution (1 mg/mL) in a weight ratio of 2:1:10, to obtain pVax-CpG/OVA, and this solution was mixed into mannan (5 mg/mL) in a 1:5 ratio with vortexing. The final product was recovered using ultracentrifugation. Decoration of mannan in MPVax enhanced the nanovaccine’s draining ability in the lymph nodes and promoted the capture of CD8^+^ DCs, whereas PLA-PEI as in inner core promoted endosome rupture and the release of antigens in the cytosol, and enhanced antigen cross-presentation. In tumor models, these nanoparticles showed superior anti-tumor effects. Glyconanoparticles also enhanced DC targeting, which can be due to MR- and DC-SIGN-mediated endocytosis, as confirmed using flow cytometry. Mannan being a TLR4 agonist, associate with CpG, a TLR9 agonist, and enhance BMDC maturation [234].

#### 9.1.5. Glycan Conjugated PLGA Nanoparticle

In the cytosol, Poly(D, L-lactic-co-glycolic acid (PLGA) particles release the endosomal payload, which is then steered towards MHC class I presentation. In another study performed by Shen, H., et al. the encapsulation of an antigen in a PLGA particle was observed to result in a two-fold increase in cross-presentation as compared to the soluble antigen [235]. The surface charge of NP is also a crucial factor to consider. For example, the negative charge on PLGA NPs confines their interactions with the negatively charged cell membrane and the intracellular uptake, restricting efficient antigen delivery into DCs [236]. 

Cancer cell membrane-coated and adjuvant-loaded PLGA-NPs with mannose modification were prepared by Yang and colleagues using an oil-in-water emulsion. In brief, PLGA polymer dissolved in dichloromethane (DCM) with added R837 agonist of TLR7 was added dropwise into 10 mL of deionized water and stirred overnight using a magnetic stirrer to evaporate the DCM. Larger particles were removed by centrifuging the particles at 4500 rpm for 20 min. The NPs were recovered after centrifugation at 12,000 rpm and re-suspended in distilled water. A B16-OVA membrane was produced by six rounds of liquid nitrogen freezing. Finally, membranes were collected by centrifuging at 14,800 rpm for 10 min. A single cold PBS wash was performed on the resulting packed B16-OVA membrane. Finally, B16-OVA membrane in 1X PBS and PLGA NPs were mixed at 4 °C for overnight. Mannose-modified B16-OVA membrane was prepared using the lipid anchor method in which B16-OVA membrane-NPs were stirred for an hour with DSPE-PEG-Man (0.1 mg/mL) and a long carbon–hydrogen was chain of DSPE-PEG-Man automatically inserted in the membrane. Mannose modification of B16-OVA-NP encapsulating TLR7 agonist R837 results in enhanced uptake and BMDC maturation. Mannose modification also enhances the MR-mediated cellular uptake of these particles by macrophages, resulting in a tumor-specific immune response [237]. Another strategy with HA-modified cationic lipid–PLGA hybrid nanoparticles proved to be a promising nanovaccine for inducing robust cellular and humoral response. These particles were developed by first synthesizing cationic lipid membranes made of 1,2-dioleoyl-3-trimethyl ammonium-propane DOTAP-PLGA NPs encapsulating the OVA antigen with a HA coating using the double emulsion (w/o/w)/solvent evaporation method. Here, OVA was dissolved in an aqueous solution. After the addition of 1 mL of DCM containing 30 mg PLGA and 6.5 mg DOTAP, it was then sonicated in an ice bath using a microtip probe sonicator. The primary emulsion obtained was then emulsified into a secondary aqueous phase 5 mL of 2% PVA in water for the formation of a secondary emulsion. From this resultant emulsion, DCM was evaporated under magnetic stirring. Resultant particles were washed in water by centrifugation and suspended in HA solution for 4 h for coating the surface of the NPs. Finally, HA-coated NPs were washed with water by centrifugation. The HA coating of DOTAP-PLGA NPs improves the cellular uptake of these particles, which is due to HA and CD44 receptor-mediated endocytosis. Enhanced activation of DCs and upregulation of MHC, costimulatory molecules, and cytokines were also found. These particles also enhance antigen-specific CD4^+^ and CD8^+^ T cell responses [238]. 

#### 9.1.6. Glyco-Clusters

Glycoclusters are also used to target DCs and can be used as a GNV. Srinivas and colleagues have synthesized a glycocluster–Melan-A/MART-1 melanoma antigen conjugate for DC targeting. For the construction of glycoclusters, first glycosynthons were synthesized by allowing oligosaccharide to react with α-glutamyl-β-alanyl benzyl ester in the presence of imidazole, which results in the formation of glycosylamine derivative which was stabilized by an in situ intramolecular acylation using Benzotriazol-1-yloxy-tris(dimethylamino)phosphonium hexafluorophosphate (BOP). Further, hydrogenolysis of the benzyl ester gave the desired glycosynthons in a larger quantity. The following oligosaccharides (lactose, dimannose, Le^a^, and Le^X^) were used in the preparation of glycosynthons. Further, coupling of glycosynthons to peptide-oligo K (Melan-A(16-40)-oligo K containing promiscuous CD8^+^ ((26-35) A27L analogue epitope) was performed with amide coupling reagents. Glycosynthon, 2-(1H-benzotriazol-1-yl)-1,1,3,3-tetramethyluronium hexafluorophosphate (HBTU) and 1-hydroxybenzotriazole (both in 0.01 mmol), 4Å molecular sieve beads (25 mg) in N-methylpyrrolidone (NMP, 1 mL) were kept under stirring conditions to form a mixture under an N_2_ atmosphere. Melan-A-oligo K peptide (0.0015 mmol) in NMP (200 µL) and 2M *N,N*-diisopropylethylamine in NMP (20 µL) was added to this solution and stirring continued for an additional 6 h. Further, molecular sieve beads were removed and 10 volumes of tert-butyl methyl ether (TBME) were added, and the crude product was precipitated and then recovered by centrifugation. The pellet was then dissolved in water and passed through the Biogel P-4 column and eluted in 50 mM acetic acid. Fast-eluting sugar-containing fractions were pooled, dialyzed, and lyophilized to yield glycocluster–Melan-A conjugate as a white fluffy powder. Dimannoside and Lewis–Melan-A conjugates bind with high affinity to MR and DC-SIGN. The DC targeted dimannoside and the Lewis–Melan-A conjugates results in efficient presentation of antigen and elicits a CD8^+^ T-lymphocyte response [239,240].

The fabrication strategies of the GNVs mentioned above and their associated benefits have been summarized in Table 4.

**Table 4:** GNV fabrication strategy and their benefits in anti-tumor immunotherapy.

GNVs	Fabrication Strategy Used to Prepare GNVs	Benefits of GNV Mediated Targeting of DCs	Reference(s)
Glyco-liposome	Thio-activated glycans (Le^x^ and Le^b^) were coupled to liposomes encapsulating OVA/MART-1 peptides via thiol–ene reaction with maleimide groups of MBP-PE.	Enhanced binding and internalization by human DC-SIGN-expressing BMDCs; 100-fold efficient antigen presentation was observed in the presence of LPS.	[158]
	Inclusion of TLR ligands (MPLA, Pam3CysSK4, R484, and Poly I:C) in glycan-modified liposomes encapsulating gp100 antigenic peptide	Inclusion of TLR4 ligand MPLA induced DC maturation, pro-inflammatory cytokine production, and significantly enhanced cross-presentation.	[195]
	Inclusion of αGC as NKT cell activator with palmitoyl-gp100/MART-1 antigen and lipo-Le^Y^ in a single liposome.	Enhanced uptake of glycoliposome by moDC, dermal DC, and LC, and induction of strong CD8^+^ and iNKT cell activation.	[228]
pH-sensitive glycan-modified liposomes	Glycan-modified pH-sensitive polymers were designed. Polysaccharide, such as dextrans, curdlan, and mannan, modified with 3-methylglutaric anhydride (MGlu) to form MGlu–Dex, MGlu–Curd, and MGlu–Man used in the preparation of pH-sensitive liposomes. Mannose modification of MGlu–Curd was carried out and used in the preparation of pH-sensitive liposomes.	Glycan-modified pH-sensitive liposomes showed maturation of DCs, targeting of CLR on APCs, cytosolic delivery of antigens, and antigen-specific cellular immune response. Mannose-functionalized curdlan derivatives incorporated in antigen-loaded liposomes showed superior pH-sensitivity than original curdlan derivatives.	[122,206,207]
Glyco-dendrimer	Branched PAMAM dendrimers used as a scaffold for gp100 long peptides and ligand Le^Y^ (for DC-SIGN and Langerin targeting) for preparing multivalent glyco-dendrimer.	Dual targeting (DC-SIGN and Langerin) by glyco-dendrimers resulted in enhanced internalization and gp100-specific CD8^+^ T cell activation.	[233]
Glyco-nanoparticles	The PLA-PEI inner core (PVax) was synthesized through nanoprecipitation. The OVA and CpG were added to PVax and mixed into mannan in a 1:5 ratio to obtain mannan-modified polymeric NPs (MPVax).	Mannan in MPVax enhances draining ability in lymph nodes and capturing by CD8^+^ DC, and promotes DC activation. The PLA-PEI enhances antigen endosome escape to promote cross-presentation.	[234]
Glycan-conjugated PLGA nanoparticle	The PLGA-NPs with the TLR7 agonist were made using an oil-in-water emulsion method and then mixed with B16-OVA membrane. Mannose-modified B16-OVA-NPs were prepared with a lipid anchor in the presence of DSPE-PEG-Man.	Mannose modification of B16-OVA-NP with TLR7 agonist R837 results in enhanced uptake and BMDC maturation. Mannose modification also enhances the MR-mediated cellular uptake of these particles by macrophages.	[237]
	Cationic lipid membranes composed of 1,2-dioleoyl-3-trimethyl ammonium-propane DOTAP-PLGA NPs encapsulating OVA antigen with HA (HA-DOTAP-PLGA NPs) coating using double emulsion (w/o/w)/solvent evaporation method.	The HA coating of DOTAP-PLGA NPs improves the cellular uptake of these particles, which is due to HA and CD44 receptor-mediated endocytosis. Enhanced activation of DCs and upregulation of MHC, costimulatory molecules, and cytokines was also found. These particles also enhance antigen-specific CD4^+^ and CD8^+^ T cell responses.	[238,241]
Glyco-cluster	Glyco-cluster–Melan-A conjugates were prepared by coupling glycosynthons. Oligosaccharyl-pyroglutamyl-*β*-alanine derivatives containing dimannoside (Manα-Man6) or Lewis antigens (Le^a^ or Le^x^) were coupled to Melan-A(16-40) peptide.	Dimannoside and Lewis–Melan-A antigen conjugate showed enhanced binding to MR and DC-SIGN. The DC targeted with these conjugated showed efficient presentation of Melan-A antigens and CD8^+^ T cell response	[239]

## 10. Where Are We Now and What Are the Lacunae in the Knowledge for Developing the GNVs?

Glyconanoparticles are excellent tools for mimicking carbohydrate presentation at the cell surfaces, hence, opening many opportunities in the field of nanomedicine [242]. Various cross-presentation studies have been performed in OT-I (CD8) and OT-II (CD4) TCR transgenic lines. Increased levels of IL-12p70 and IFN-γ are considered as the indicators of cross-presentation. The present-day cues and leads for the validation of the concept are insufficient. Besides tricking the immune system into recognizing the GNVs as pathogen-like foreign material, it must educate the immune system to recognize the antigens present on live tumor cells, thereby aiding eradication. Recent reports demonstrate successful cross-presentation of antigens on live cells [243,244]. Furthermore, the antigens from living cells are observed to cross-present more efficiently than the dead cells. Thus, GNV-directed cross-presentation of live tumor cells seems feasible in the near future. The first GNVs protected with self-assembled monolayers of different tumor-associated-carbohydrate immunogenic peptides were claimed to be prepared by García and colleagues as anticancer vaccines; a study of immunologic responses of these constructs was alleged to be in the process of being carried out [245].

The extensive research on cross-presentation has placed the limelight on its promising prospect as potential cancer therapeutic; however, the major understanding of fundamental questions for in vivo applications still remains unanswered, instigating unreciprocated outcomes. The contrivances that make CD8^+^ DCs proficient for performing cross-presentation and the factors behind this observed dogmatism remain to be understood. The biasness of antigens cross-presented via phagosome-to-cytosol routes over the vacuolar or cathepsin S-vacuolar pathways, and the probable mechanism of how the ER components are formed is unclear. The questions, such as which stage of cancer can be treated with GNVs and if there are any collateral damages, are unaddressed. Whether the adaptive response can be provoked by employing the glycoconjugates intended to maximize the formation of glycan epitopes remains to be examined [246]. Besides this, to what extent the cross-presentation contributes to the CD8^+^ T cell response in vivo and whether any other microenvironment stimuli are required or not needs to be analyzed. These lacunae in knowledge needs to be addressed to practice and improvise GNVs.

## 11. What Do We still Need to Do and Where Are We Going Next?

The OVA, chicken egg protein, and CD8^+^ T cell epitope of OVA are the most commonly used antigens to study the cross-presentation both when coated and encapsulated in polymer particles. Even though these antigens can induce the prophylactic CD8^+^ T cell response, these studies may or may not imitate the desired practical outcome. The bias over the selection of antigens used to study cross-presentation needs to be minimized. This can be achieved by using specific tumor antigens to study cross-presentation instead of using model antigens. With this being said, the cost of the TAAs is a limiting factor for conducting such studies. The cell-associated OVA antigen has been reported to cross-present more efficiently than soluble ovalbumin in vivo [176]. Considering this observation, the use of tumor lysate instead of specific TAAs to cut short on expenses could be a foreseen approach. However, the tumor lysates are susceptible to rapid clearance and require new experimental approaches. The validation of GNVs for the cross-presentation application to achieve the desired levels of CD8^+^ T cell response remains to be performed. Apart from this, the experimental methods to quantify the contribution of cross-presentation in specific immune responses still need to be confirmed. The current experiments are performed using the mouse DC–cell lines; however, these outcomes cannot be extrapolated into clinical applications as the human and mouse DC subsets are dissimilar and generate a dissimilar immune response. Furthermore, the million years of independent evolution that has contributed to the distinctions in their immune systems cannot simply be ignored [247]. Herein, the cross-presentation-defective mice are, so far, the best thought system to study and understand cross-presentation [248]. In 2003, Met, Ö. and colleagues demonstrated that the OVA_257–264_-peptide (also known as SIINFEKL)-loaded DCs were able to prime and activate MHC-class I-restricted T cells more efficiently than OVA protein-loaded cross-presenting DCs [249]. The characteristic features of SIINFEKL peptides that favor efficient cross-presentation, can be harnessed for selecting the tumor antigenic peptide for effective cross-presentation. The comparative studies between the SIINFEKL and various TAAs may help us recognize the specific pattern required for effective cross-presentation. Imitating the anti-pathogen CD8^+^ CTL to tailor cross-presentation-mediated CTL response is essential. The composition of the glycan moiety of pathogens is a deciding factor for the triggered immune response. The polysaccharide’s multivalent display or other modifications have the potential to specifically tailor the immunological outcome. Different compositions of glycan found on the pathogens mediated different kinds of immune response; for example, fucose, which differs from mannose only in a methyl group, extends a disparate immune response on interaction with CLRs [209]. The ability of the DCs to interact with the heterogeneous glycans versus the homogenous glycans needs to be investigated for assured and enhanced cross-presentation. Apart from this, which of the DCRs participates best in cross-presentation and what proportion of this receptor governs what percentage of cross-presentation still needs to be investigated. Establishing the order of different types of nanocarriers based on the efficiency to cross-present are some of the topics for future research. The oral, nasal and subcutaneous routes of pathogen delivery have been demonstrated to trigger effective CTL in anti-pathogen responses. The mode of delivery of the GNVs would be a deciding factor for the accomplishment of the CTL response. However, targeting the subcutaneous DCs for cross-presentation is considered by far the most effective approach. For this, techniques to deliver the GNVs specifically to the DCs are required and may be achieved through injecting the GNVs into the skin DCs. Here, in our opinion, a painless technique, such as a ‘microneedle’, that works by imitating mosquito bite may perhaps serve the clinical utility to deliver the drug [250,251,252] to CD1a^+^ and CD14^+^ dermal DCs (dDCs) located in human skin-draining lymph nodes that harbor effective T cell stimulating capacities [90,253]. The antigens from apoptotic cells have been reported to get cross-presented. This finding should be explored for supplementing the cross-presentation therapy with other anti-cancer therapies, such as chemotherapy and irradiation, that result in the generation of apoptosised tumor cells. Targeting such apoptosised tumor cells for cross-presentation may help render sustained memory response along with CTL against the recurring cancer cells. Furthermore, finding ways to utilize the cross-presentation abilities of all the potential APCs along with DCs is the next anticipated research goal. New immunotherapeutic techniques are being studied and developed by the research community for cancer treatment. Teran-Navarro et al., through their study, demonstrated that gold glyconanoparticles combined with certain peptides of Listeriolysin O, a bacterial toxin, can be used for bladder tumor treatment [254]. Another approach is to use near-infrared probe conjugated glyconanocarriers for targeted drug delivery [255]. Partially-oxidized acetylated dextran nanoparticles combined with cytotoxic T lymphocyte peptide and immune targeting adjuvant R837 are also being developed as a nanocarrier for encapsulating hydrophilic peptides, as acetylated dextran nanoparticles cannot encapsulate them [256].

## 12. Challenges Associated with Developing GNVs as Cancer Immunotherapy

The inherent property of cancer cells is that they stimulate genetic modification and, thus, cause alterations in the expression of the antigen. Furthermore, considering different antigens or different forms of antigens may end up engaging different receptors present on cells; therefore, the fate of targeting different receptors may land antigens into distinct subcellular compartments, or this may also influence the state of the APCs in a way that can affect the pathway used for antigen cross-presentation. In addition to this, the mechanisms underlying GNVs that target TACAs could be uncertain. The cross-presentation does not entirely contribute to priming the CTLs but, in addition to this, it may induce cross-tolerance [91,257]. Thus, minimizing the cross-tolerance is the next hurdle to overcome. Along with this, maximizing the efficiency of cross-presentation is the next associated challenge [258]. Other associated challenges should also be considered while designing the successful GNV-mediated cancer immunotherapy [259]. 

Several immune checkpoints are up-regulated as a result of genetic and epigenetic alterations produced by cancer’s high mutation rate [260]. Using these checkpoints, tumor cells suppress anti-tumor immunity and promote the establishment of an immunosuppressive environment [261]. The CTLA-4 and PD-L1 immune checkpoints limit T cell cytotoxicity and prevent tumor cells from being eliminated. The CTLA-4, a cell surface molecule present on T lymphocytes, binds to its ligands (B7.1 and B7.2) on APCs and further serves as a negative regulator of T cells [262]. Similarly, the PD-L1 molecule on tumor cells functions as a ligand for the PD-1 molecule on T cells. As a result of their interaction, T cell proliferation is inhibited [263,264]. Due to the immunosuppressive nature of immune checkpoints, blocking them is essential, and the development of antibodies against PD-L1, PD-1, and CTLA-4 has made it feasible [265,266,267]. In combination with these antibodies, radiofrequency ablation (RFA), along with hemin and LOX co-loaded CaCO_3_-encapsulated PLGA nanoreactors, can enhance anti-tumor immunity and inhibit tumor growth [268]. Another strategy for improving the efficacy of immunotherapies is vitamin C supramolecular hydrogel, as vitamin C has been shown to promote cancer immunotherapy [269].

### Glycan-Lectin Interaction in the Induction of Immunosuppressive TME

In recent times, tumor glycosylation has also been considered as a new class of immune checkpoint molecule. Tumor cells present an altered glycan coat on their surface compared to the normal healthy cells. These aberrant glycans are recognized by immune lectins, which are able to decode these glycan signatures. This glycan–lectin interaction often induces the induction of immunosuppression, which further promotes tumorigenesis [259]. This induction of immunosuppression is prompted by overexpression of self-glycans (for example sialic acid) which are termed as ‘self-associated molecular patterns’ (SAMPs). Cancer cells take advantage of these glycosylation modifications by masking them as host-like and seizing control of the immune system for their own benefit [217]. Tumor-associated glycans, such as sialylated structures, Tn antigen, and Lewis antigens, are examples of glycans present as membrane-bound or tumor-secreted proteins [270]. Hijacking of glycan responses can promote immune evasion by altering APC functions, encouraging the differentiation of tumor-associated or anti-inflammatory M2 macrophages or by modifying the T cell differentiation process and NK cell activity. Therefore, it is very necessary to understand the glycan signature of tumor cells (referred to as glyco-code) and how these glycan–immune lectin interactions can drive the immunosuppression in TME. 

Several studies have shown that immune cells express various lectin receptors, such as sialic acid-binding immunoglobulin-like lectins (SIGLECs), DC-SIGN, and MGL; these lectin receptors, after binding with tumor glycans, mediate immune suppression. Specific examples, such as hyper-sialylation of tumor cells increased the expression of SIGLECs’ ligands sialic acid on their surface. The SIGLECs binding to the sialic acids induces a strong inhibitory function which leads to immune suppression [238]. Perdicchio and colleagues showed that hyper-sialyation of melanoma cells was associated with an increase in tumor growth in vivo, which was associated with enhanced Treg cells, a reduced number of effector T cells, as well as reduced NK cell activity [271]. Sialylated glycans present on tumor cells can directly interact with SIGLEC7 and SIGLEC9 expressed on NK cells, and reduces the activity of these immune cells [272]. Furthermore, SIGLEC15, another SIGLEC family member, was found largely upregulated in human cancer cells, tumor-infiltrating myeloid cells, and inhibited T cell response. Genetic ablation or antibody-mediated blocking of SIGLEC15 improved the anti-tumor immunity and blockade of the tumor growth in mouse models [273]. 

Galectins are the family of soluble *β*-galactoside binding lectins that have taken a leading role in current times due to their involvement in cancer, prognostic value, and the targeting of these lectins for therapeutic purposes [274]. Galectins are expressed by a wide range of tumors, and their overexpression is correlated with the aggressiveness of tumors and further conversion into the metastatic form [275]. In recent times, galectins have emerged as novel regulatory checkpoint molecules that are involved in impaired effector T cell functions in various ways, as follows: by inducing T cell exhaustion, restricting its survival and, by favoring Tregs expansion, deactivation of NK cells and instructing differentiation of myeloid cells towards the immunosuppressive phenotype [276]. Galectins (galectin-1, -3, and -9) are highly studied in the context of cancer. Galectin-1 contributes immune evasion and suppression of immune surveillance through various mechanisms which include galectin-1, as well as other soluble factors secreted in TME, which induce normal DCs to tolerogenic DCs conversion; therefore, it suppresses T cell response. Galectin-1 also promotes apoptosis of Th1 and Th17 cells [277,278]. Inhibition of galectin-1 in TME enhances CD4^+^ and CD8^+^ T cell functions [279]. Similarly, normal immune surveillance of NK cells is also suppressed by malignant glioma cells through the overexpression of galectin-1, and further suppresses galectin-1 and restores the normal surveillance of NK cells and eradicates glioma cells [280]. Galectin-3 is also involved in silencing NK cell activity. Galectin-3 binds with the NK cell receptor D (NKG2D)-binding site of MHC class I-related chain A (MICA) via interaction with poly-*N*-acetyllactosamine present on core2 *O*-glycans and, thereby, reduces the affinity of MICA with NKG2D [281]. The T cell Ig and mucin domain 3 (TIM3) was expressed on Th1 and CD8^+^ T cells. Galectin-9 was identified as a ligand for TIM3. Galectin-9 binds to the TIM3 IgV domain in a glycan-dependent manner and further initiates a suppressive function in T cells. Furthermore, overexpression of TIM-3 ligand galectin-9 enhances the frequency of myeloid-derived suppressor cells and promotes CD8^+^ T cell exhaustion [282,283]. Therefore, this enhances the secretion of galectins by tumors and creates an immunosuppressive TME affecting many immune cells. 

Antigens presented by MHC class I and II molecules have the capability to retain some post-transcriptional modifications, such as phosphorylation and glycosylation; therefore, an additional level of neo-antigenicity is added to tumor-specific peptides presented by MHC class I molecules. Furthermore, T cells with a TCR specific for glycopeptides have been identified, and these TCRs recognized only the glycosylated form of peptides [284,285]. Therefore, the nature of tumor-infiltrating T cell repertoire and tumor cell expressing glycopeptide–MHC-I complexes require further investigation for the identification of unique tumor-specific epitopes and their glycopeptide-specific T cells [286].

**Figure 4:**
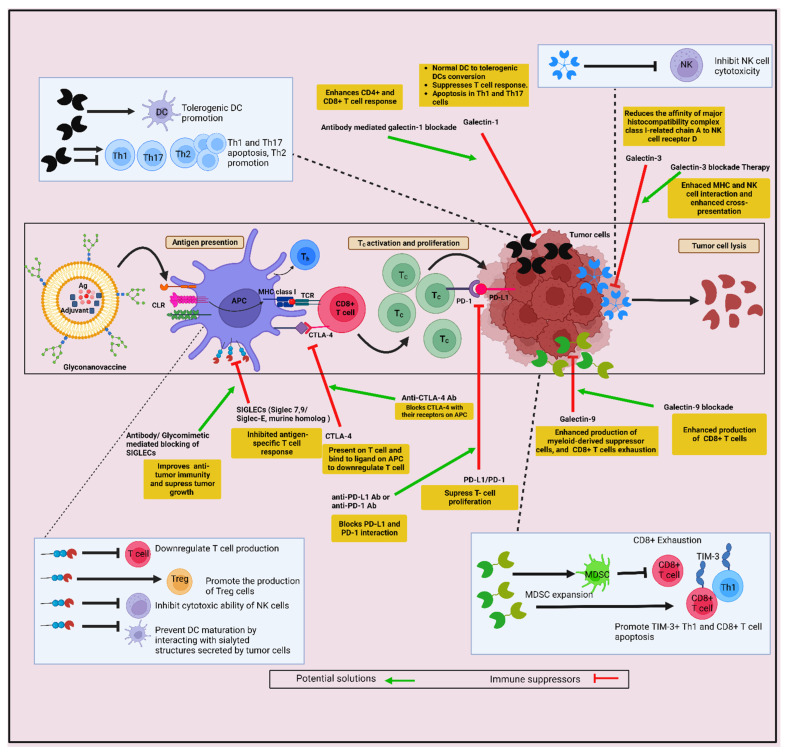
Strategies to enhance the anti-tumor response of glyconanovaccines (GNVs). Generation of a large number of tumor antigen-specific T response will be induced through the injection of GNVs to the skin DCs. Targeting of the GNVs to the specific DC subset is achieved by surface modification with specific glycans to target CLRs on DCs for the internalization and subsequent tumor antigen presentation and maturation of DCs. This leads to the priming and activation of T cells that are specific for tumor-antigens, creating a large pool of effector cytotoxic T cells that are capable of moving toward tumors, penetrating them, and ultimately killing tumor cells once they have been recognized. The anti-tumor response will be enhanced by the administration of checkpoint inhibitors, such as anti-CTLA-4, anti-PD-1 antibodies, glycomimetic/anti-Siglec antibodies, and further inhibition of galectins (galectin-1, -3, and -9). (Created with BioRender.com accessed on 27 September 2022).

Therefore, a better understanding of the nature of this code–decode system, i.e., the glycan language of the tumor (glyco-code) and the lectins present on the immune cells (decoder), will aid in designing better anti-tumor immunotherapies. The challenges associated with the development of the GNVs and their possible solutions are described in Figure 4. 

In conclusion, adequate preclinical and clinical research needs to be performed to validate the concept of GNVs. Advances in understanding of innate immunity over the last decade have fueled interest in how DCs can be exploited to develop immunotherapies. It is a reasonable expectation that GNVs targeting DCs via antigen cross-presentation will boom in the near future. Glycan-conjugated nanovaccines bearing TAAs profoundly influence CTL immunity, most notably by facilitating the cross-presentation of the antigens by DCs. Although the evidence for the therapeutic effectiveness of this strategy is still expanding, the development of glyco-nanovaccine-mediated anti-tumor immunotherapy requires more research to become the leading anti-tumor immunotherapy in the future. 

## Figures and Tables

**Table 3:** Immune response due to pathogens and imitative approaches.

	Pathogens	Imitative Approach	Reference(s)
Size	Viruses (20–200 nm)	DC-targeted nanoparticle of the same size as viruses (20 nm to 200 nm)	[214,215]
Interactions	Interactions between the carbohydrates present on the pathogen surface and the APC receptor	Nanoparticle coated with glycan interacts with specific CLRs	[216,217]
Escaping endo/lysosomal trafficking pathways into the cytosol	Using pH-dependent mechanisms	pH-responsive endosomal escape	[218]
Immune cell recruitment	Achieved through cytokine secretion	Increased cytokine secretion and immune cell recruitment on administration	[219]
Surface properties	Pathogen-associated molecular patterns (PAMPs)	Glycan-coated nanoparticles	[78,220]

## Data Availability

Not applicable.
